# Supplementary material for: Single cell spatial analysis reveals inflammatory foci of immature neutrophil and CD8 T cells in COVID-19 lungs
Source: Nat Commun. 2023 Nov 8;14:7216. doi: 10.1038/s41467-023-42421-0 (PMC10632491; doi:10.1038/s41467-023-42421-0)
Supplement: Supplementary file 1 — Supplementary Information [file 41467_2023_42421_MOESM1_ESM.pdf]

Oxford_ID	Age	Sex	CT days before tissue sampling (death)	CT findings	ICU (days)	Days from first symptoms	Days in hospital before death	Medical History	Symptoms	leucocyte 10E9/L	Neutrophils 10E9/L	Lymphocytes 10E9/L	CRP (mg/dL)	Ferritin (ng/ml) NV:15-150	D-dimer (ng/ml) NV: 150-500	Troponina T (ng/L) NV≤14	Pro-BNP (pg/ml) NV≤222	Drugs during hospital stay
CUN20	85-90	F	3	GGO, CONS,AB,CPp,PE	0	10	3	AD	Fever Dyspnea Cough	6.90	5.95	0.60	1.00	543	550	445	2090	HC, AZM, HEP
CUN17	75-80	M	4	GGO, AB,CPp,PE	0	8	5	HBP, DM2, AD, HF	Dyspnea	6.20	5.22	0.45	35.62	7590	330	407	33600	HC, AZM, LPV/r, HEP
CUN19	65-70	M	2	GGO, CPp, CONS, AB	12	13	12	HBP, DM2, Obesity, DLP, COPD	Fever Dyspnea Myalgia	5.90	5.68	0.10	51.73	2084	100	18	2530	HC, AZM, CCS, LPV/r, Betaferon,
CUN8	75-80	M	2	GGO, AB, CONS, PE	0	8	3	HBP, COPD, DM2, DLP, CKD, HF, IC, ICD,	Fever Dyspnea Cough	9.90	8.74	0.48	21.55	1140	480	164	4390	HC, AZM, HEP
CUN9	70-75	F	12	GGO, CONS, AB, PE	6	15	14	HBP, CKD, KT, Lymphoma, Obesity	Fever Cough	13.20	12.47	0.23	0.14	1836	190	6052	1530	HC, AZM, LPV/r, CCS, HEP
CUN10	75-80	M	20	GGO, CPp	18	25	21	HBP	Fever Cough	11.00	9.84	0.45	0.38	2200	200	650	3240	HC, LPV/r, CCS, HEP
CUN11	90-95	M	11	GGO	0	14	9	HBP, DM2, COPD, DLP, Obesity, Afib, HF,	Cough	9.10	8.26	0.39	4.64	1440	270	24	2030	HC, AZM, CCS, HEP
CUN4	80-85	M	11	GGO, RETp (Gen), PE	0	17	12	HBP, LC, COPD	Fever Dyspnea Cough	12.00	10.99	0.24	0.43	1016	220	27	2510	HC, AZM, CRO, CCS, LPV/r
CUN6	60-65	F	4	GGO, CONS	23	29	27	HBP, DM2, Obesity, DLP, HF, IC	Fever Dyspnea	4.70	3.58	0.85	18.94	1040	650	503	7010	HC, AZM, CRO, CCS, LPV/r
CUN14	85-90	M	42	GGO	7	19	12	HBP	Fever Dyspnea Cough Diarrhea	7.90	7.24	0.34	0.83	2680	190	27	2220	HC, AZM, CCS, Tocilizumab
CUN16	30-35	F	14	GGO, CPp, CONS	12	29	27	KT, Lymphoma, NH	Fever	17.80	15.61	0.25	0.46	9415	50	39	3670	HC, AZM, CCS, Tocilizumab,
CUN5	60-65	F	29	GGO, CONS	19	46	43	HBP, HF, Afib, Lymphoma	Fever Cough Odynophagia Diarrhea	3.50	2.19	0.91	9.17	13918	130	44	3940	HC, AZM, CCS, Tocilizumab, Remdesivir

Supplementary Table 1A. Clinical data of patients. Medical history: Arterial hypertension (HBP); Chronic Obstructive Pulmonary Disease (COPD); Rheumatoid Arthritis (RA); Alzheimer's Disease (AD); Diabetes Mellitus type 2 (DM2); Atrial fibrillation (Afib); Restrictive cardiomyopathy (RCM); Dyslipidemia (DLP); Chronic Kidney Disease (CKD); Heart Failure (HF); Ischemic cardiomyopathy (IC); Implantable Cardioverter Defibrillator (ICD); Cerebrovascular Accident (CVA); Kidney transplantation (KT); Follicular Lymphoma (FL). CT findings ground-glass opacities (GGO); Consolidation (CONS); "Crazy Paving" pattern (CPp); Pleural effusion (PE); Air bronchogram (AB). C-Reactive protein (CRP). Treatment (Tx): Hydroxychloroquine (HC); Azithromycin (AZM); Lopinavir/ritonavir (LPV/r); Corticosteroids (CCS); Heparin (HEP). Intensive Unit Care (ICU) days. Duration of clinical course (DCC). We confirm that Oxford_IDs are anonymous and unidentifiable IDs - noone outside a small group of researchers can identify the source of these IDs. Age for the patients have been provided in an aggregate to prevent identification.

Indication for tissue harvest	Age	Gender	Other lung disease	Histopathology of section	Study use
Localised lung cancer	70	M	None	Normal	H&E, IF and IMC
Localised lung cancer	64	M	None	Normal	H&E, IF and IMC
Localised lung cancer	68	F	None	Normal	H&E and IF
Localised lung cancer	85	F	None	Normal	H&E and IF
Localised lung cancer	73	M	None	Normal	H&E and IF
Localised lung cancer	81	M	None	Normal	H&E and IF
Localised lung cancer	73	M	None	Normal	H&E and IF
Localised lung cancer	58	F	None	Normal	H&E and IF

Supplementary Table 1B. Demographics for normal lung sections (HC) used in main study (IMC) and in supplemental studies (IF - immunofluorescence ; and H&E - haematoxylin and eosin)

	Nucleocapsid protein staining	Nucleocapsid PCR (Ct)	Histopathological state
**Cun 14**	pos	32.2	ALV
**Cun 20**	neg	30.5	ALV
**Cun 6**	neg	>36	ALV
**Cun 8**	neg	29.1	ALV
**Cun 4**	pos	27.3	DAD
**Cun11**	pos	26.8	DAD
**Cun17**	pos	30.7	DAD
**Cun9**	pos	17.8	DAD
**Cun 16**	neg	26.9	OP
**Cun 19**	neg	33.9	OP
**Cun 5**	neg	34.2	OP
**Cun10**	neg	36.0	OP

Supplementary Table 2. Immunohistochemistry for N protein, PCR for N and corresponding samples and their histopathology states

Metal	Target
141Pr	αSMA
142Nd	CD56
143Nd	Vimentin
144Nd	EpCAM
145Nd	-
146Nd	CD16
147Sm	CD64
148 Nd	Pan Keratin
149Sm	CD15
150Nd	TMPRSS2
151Eu	CD200R
152Sm	Pan membrane*
153Eu	Vα7.2
154Sm	CD45
155Gd	FoxP3
156Gd	CD4
157Gd	-
158Gd	Ecadherin
159Tb	CD68
160Gd	PF4
161Dy	CD20
162Dy	CD8a
163Dy	CD14
164Dy	CD161
165Ho	ACE2

Metal	Target
166Er	-
167Er	Granzyme B
168Er	Ki67
169Tm	Collagen I
170Er	CD3
171Yb	Histone 3
172Yb	CD31
173Yb	CD45RO
174Yb	-
175Lu	-
176Yb	-
191Ir	DNA dye 1
193Ir	DNA dye 3

Supplementary Table 3 Panel 1 metal-tagged antibody panel for sentinel cohort

Cell type label	Consortium led phenotype call
Myofibroblast	Myofibroblast
Fibroblast	Fibroblast
Prolif fibroblast	Active proliferating fibroblast
Blood vessels	Blood vessels including bronchial and pulmonary artery and veins
Endothelial cells	Mainly alveolar capillaries but also some blood vessels
Prolif endothelial cells	Active repairing endothelial cells
Bronchial epit	Healthy HLADR^{lo} bronchial epithelium. These are the only bronchial epithelium found in healthy lungs
Prolif alveolar epit	RAGE^{+} Type II alveolar epithelium and alveolar progenitor cells
Prolif bronchial epit	Active repairing bronchial epithelium
HLADR^{lo} bronchial epit	HLADR^{lo} bronchial epit
HLADR^{hi} bronchial epit	Inflamed bronchial epithelium
Mono PAI-1 ADJ	Monocytes adjacent to PAI-1 expressing cells, typically activated/inflamed endothelium
Neut CD8 ADJ	CD15^{hi} Neutrophils adjacent to CD107-GZB^{+}CD8 T cells
CD15^{lo} iNeut	Least immature neutrophils
CD15^{mid} iNeut	Mid immature neutrophils
CD15^{hi} iNeut	Most immmature neutrophils
IFN-β ^{hi} CD10^{+} cells	Possible mature neutrophils (but no CD15 expression)
Mac1	CD14^{hi} IFN-β IFN-γ^{+} 'transitional' macrophage (Least mature macrophage)
Mac2	CD14 ^{mid} Macrophage (Mid Mature macrophage))
Mac3	HLADR^{lo} CD14^{-} mature Macrophage (Most Mature macrophage)
Mono3	Mixed population of monocytes - some classical CCR2 ^{hi} CD68^{lo} monocytes , some intermediate(CD14^{hi}CD16^{hi}) , some Ki67 $^{lo/mid}$ monocytes (immature cycling monocytes); overall - most differentiated monocyte. 'Differentiation 'refers to differentiation to macrophage
Mono2	Classical monocytes, CCR2 $^{lo-hi}$ CD68^{lo} monocytes (mid differentiated monocyte) Ki67^{-}
Mono1	Classical monocytes, CCR2^{lo} CD68 ^{neg} monocytes (least differentiated monocyte) Ki67^{-}
Mono_CD31_ADJ	CCR2^{lo} CD68 ^{neg} monocytes (least differentiated monocyte) Ki67^{-} adjacent to endothelial cells. Some were monocytes with CD31 expression
IFN–γ hi RAGE ADJ	CD45^{+} IFN-γ ^{hi} expressing cells with no epithelial marker and high RAGE expression. Possible resident alveolar macrophage adjacent to RAGE^{+} alveolar epithelium; but no CD68 expression
CD8_CD31_ADJ	CD107^{-} CD8 adjacent to endothelial cells
CD107^{+} CD8	CD45RO effector memory cytotoxic CD8 T cells
CD107^{-} CD8	CD45RO effector memory CD8 T cells , likely exhausted cytotoxic CD8 T cell
IFN–γ^{+} CD4 T cells	CD45RO effector memory activated CD4 T cells
IFN-γ^{-} CD4 T cells	Non-activated CD4 T cells
CD107^{+}CD4 T cells	Cytotoxic CD4 T cells
IFN-γ^{hi} NK	Activated NK cells
IFN-γ^{lo} NK	NK cells
IFN-γ^{hi} MAIT cells	Activated MAIT cells
IFN-γ^{lo} MAIT cells	MAIT cells
Vα7.2lo cells	MAIT-like cells
CD8_PAI_ADJ	CD107^{-} CD8 adjacent to activated/repairing endothelial cells
Megakaryocyte	Megakaryocyte

Supplementary Table 4. Expanded description of phenotype of cells

**CYTOF (Neutrophil staining)**
**Marker (clone)-metal**
CD45 (HI30)-89Y
CD5 (UCHT2)-111Cd
CD3 (UCHT1)-112Cd
CD9 (HI91)-113Cd
CD7 (CD7-6B7)-114Cd
HLADR (L243)-116Cd
CD49d (9F10)-141Pr
CD19 (HIB19)-142Nd
CD123 (6H6)-143Nd
CD15 (W6D3)-144Nd
CD38 (HIT2)-145Nd
CD64 (10.1)-146Nd
CD11c (Bu15)-147Sm
CD16 (3G8)-148Nd
CD74 (LN2)-149Sm
CD43 (84-3C1)-150Nd
CD103 (Ber-ACT8)-151Eu
CD66b (80H3)-152Sm
BDCA2 (201A)-153Eu
CD163 (GHI/61)-154Sm
CD36 (5-271)-155Gd
CD10 (Hi10a)-156Gd
CD33 (WM53)-158Gd
CD22 (HIB22)-159Tb
CD14 (M5E2)-160Gd
CLEC9A (8F9)-161Dy
ki67 (B56)-162Dy
CD172a/b (SE5A5)-163Dy
**Marker (clone)-metal**
Siglec 8 (7C9)-164Dy
CD101 (BB27)-165Ho
CD141 (M80)-166Er
CD301 (H037G3)-167Er
CD71 (OKT9)-168Er
BDCA4 (12C2)-169Tm
CD114 (38660)-170Er
CD226 (DX11)-171Yb
CD354 (TREM-26)-172Yb
CD371 (50C1)-173Yb
CD142 (NY2)-174Yb
CD274 (29E.2A3)-175Lu
BDCA1 (L161)-176Yb
CD11b (ICRF44)-209Bi

Supplementary Table 5 Metal-tagged antibody for suspension CYTOF staining

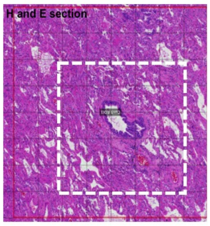

A. Analysis of overall histo-pathology features from HnE stained first slide from paraffin embedded tissue. Relevant ROI drawn (2-3 of 2x2mm per patient).

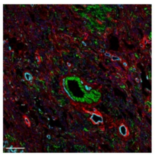

B. Staining of adjacent lung tissue section with a 37-plex metal-tagged antibody panel and acquisition by IMC (Hyperion). Individual metal intensity signals linked to x,y coordinate of ablated area are then digitised to generate an MCD image file.

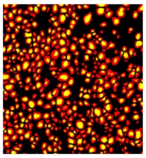

C. Segmentation of OME-TIFF (generated from MCD image file) into single cells. Cell masks and metadata (x,y location, area) are generated using Mesmer library from Deep Cell platform (Greenwald N 2022)

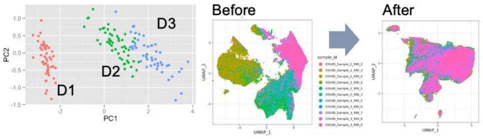

D. Signal intensity from each cell is extracted for dimension reduction and clustering using R Phenograph. Batch effect from ablation and staining days is adjusted using Harmony integration (Korsunsky I 2021).

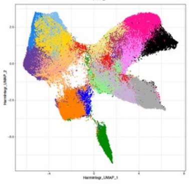

E. Annotation of clusters is performed using expression density plot, heat map analysis, Pseudotime of selected groups of clusters, cluster distribution plot, and cross checked with H&E and MCD images (Figure S7).

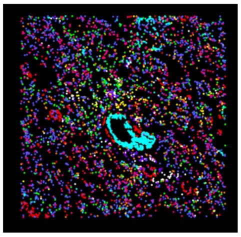

F. Creation of cell centroid map, providing identity and location for all annotated cells in a given ROI.

Spatial statistical analysis of co-locating cells

i.

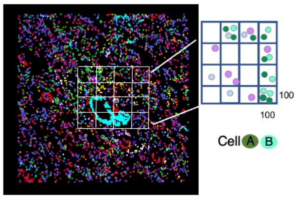

ii.

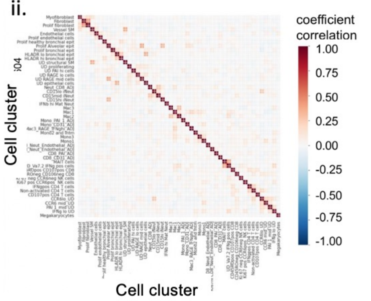

iii.

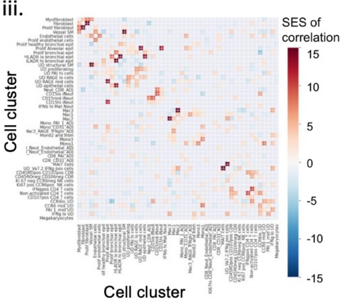

G. Step 1. Quadrat Correlation Matrix (QCM) analysis. Each ROI is first divided into 100x100um quadrats (i, only 16 quadrats out of 400 quadrats in the 2x2 mm ROI are shown, for clarity) and correlation between all pairs of annotated cells in each quadrat is analyzed using Morueta-Holme 2016 methods. Coefficients of correlation and standardized effect size (SES) for these correlations (above null distribution) and adjusted for multiple comparisons by Bonferroni correction are calculated (ii and iii). Pairs of cells that have SES with FDR q < 0.05 are submitted for cross pair correlation function (cross-PCF) analysis.

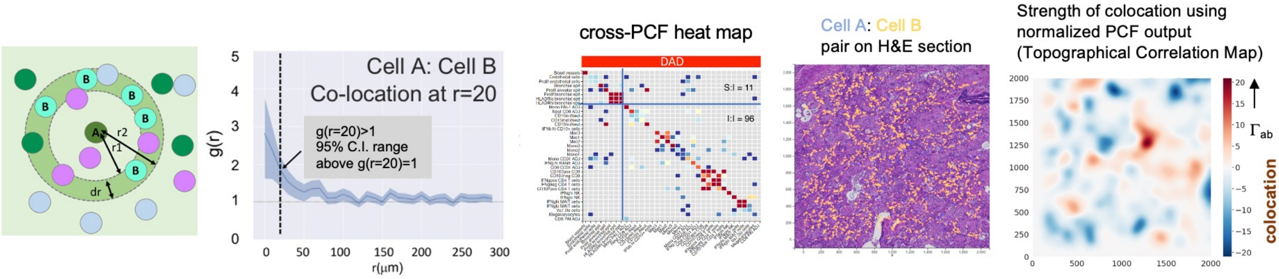

H. Step 2. Cross pair correlation function (cross-PCF) analysis. Pairs of cells which passed the significant correlation test from the QCM analysis are tested for co-location with each other. An annulus is drawn round an anchor cell of interest (cell A) at r1=20μm and r2 =30μm from the centroid of cell A. Frequency of cell B in this annulus is calculated against complete spatial randomness and a 95% confidence interval fitted for the curve for pairs of cells in a specified relevant group of ROIs. Pairs of cells with g(r=20) >1 and 95% C.I. range above g(r=20)=1 are termed statistically significantly co-located at 20μm from the designated anchor cell (Cell A). Finally, a topographical representation of statistically significant co-locating cells is generated for a given ROI using an adapted PCF, termed 'Γ_{ab}'. Γ_{ab} measures the frequency of cells in the space from the centroid of Cell A to the edge of r2.

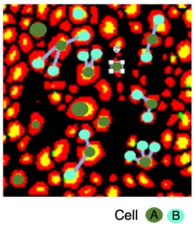

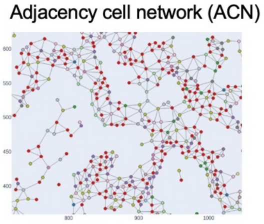

I. Step 3. Adjacent cell network (ACN). For the cell pairs that were significantly co-located, we then analyze the network of cells that are in direct physical contact with the anchor cell of interest using a boundary enhanced cell centroid map. Thus, for cell A (anchor cell) that has been found to be significantly co-located with cell B by QCM and cross-PCF analysis, we compute the proportion of cell A in the ROI that are physically in contact with cell B (ϕ_{AB} , see methods). In this exemplar plot, there are 12 cells of type A in the ROI; and 8 (67%) have contact with at least one cell of type B.

Supplementary Fig. 1. Sequential steps in overall segmentation and spatial analysis pipeline with description of each step

A.

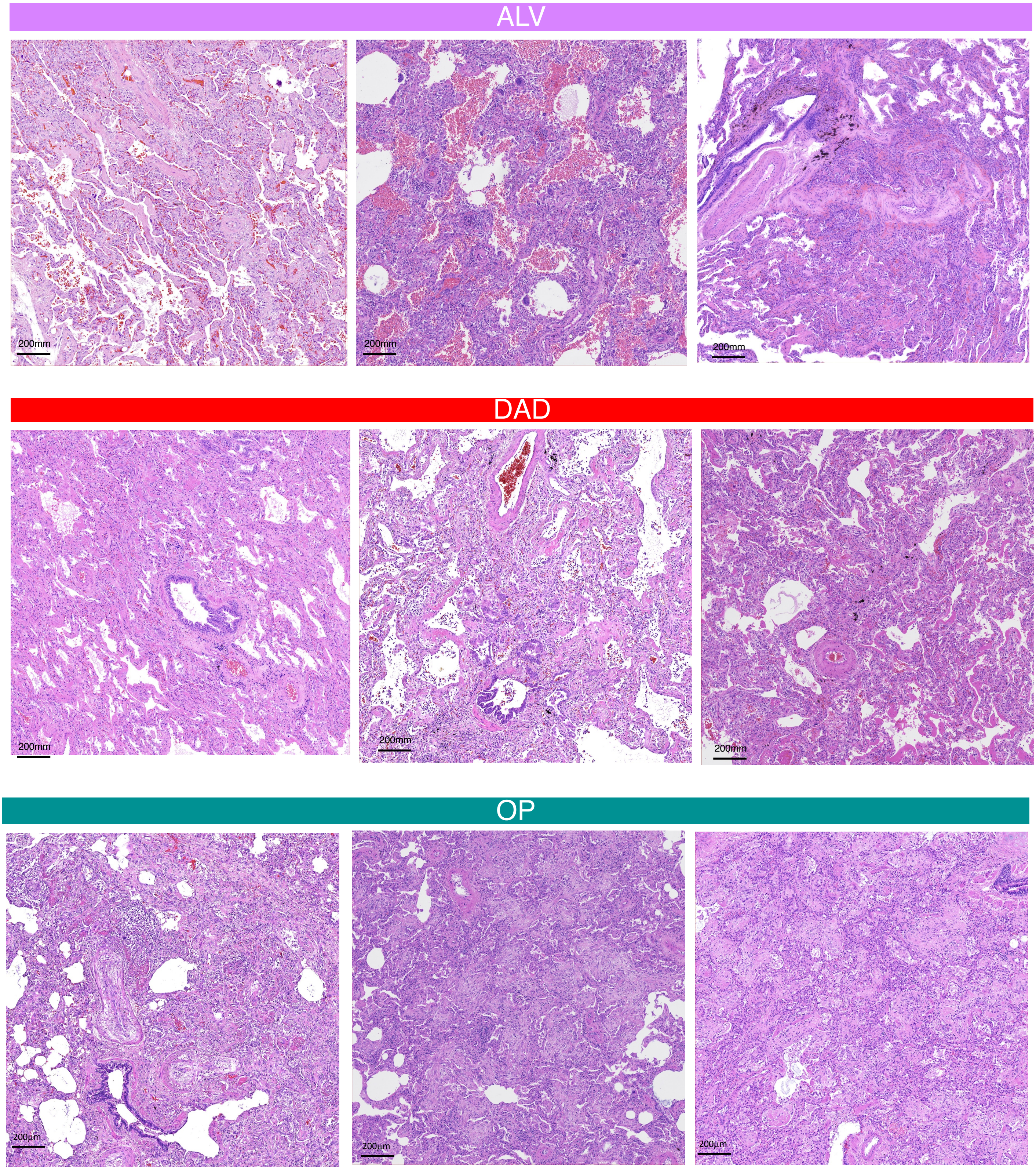
ALV
200mm
200mm
200mm
DAD
200mm
200mm
200mm
OP
200µm
200µm
200µm

B.

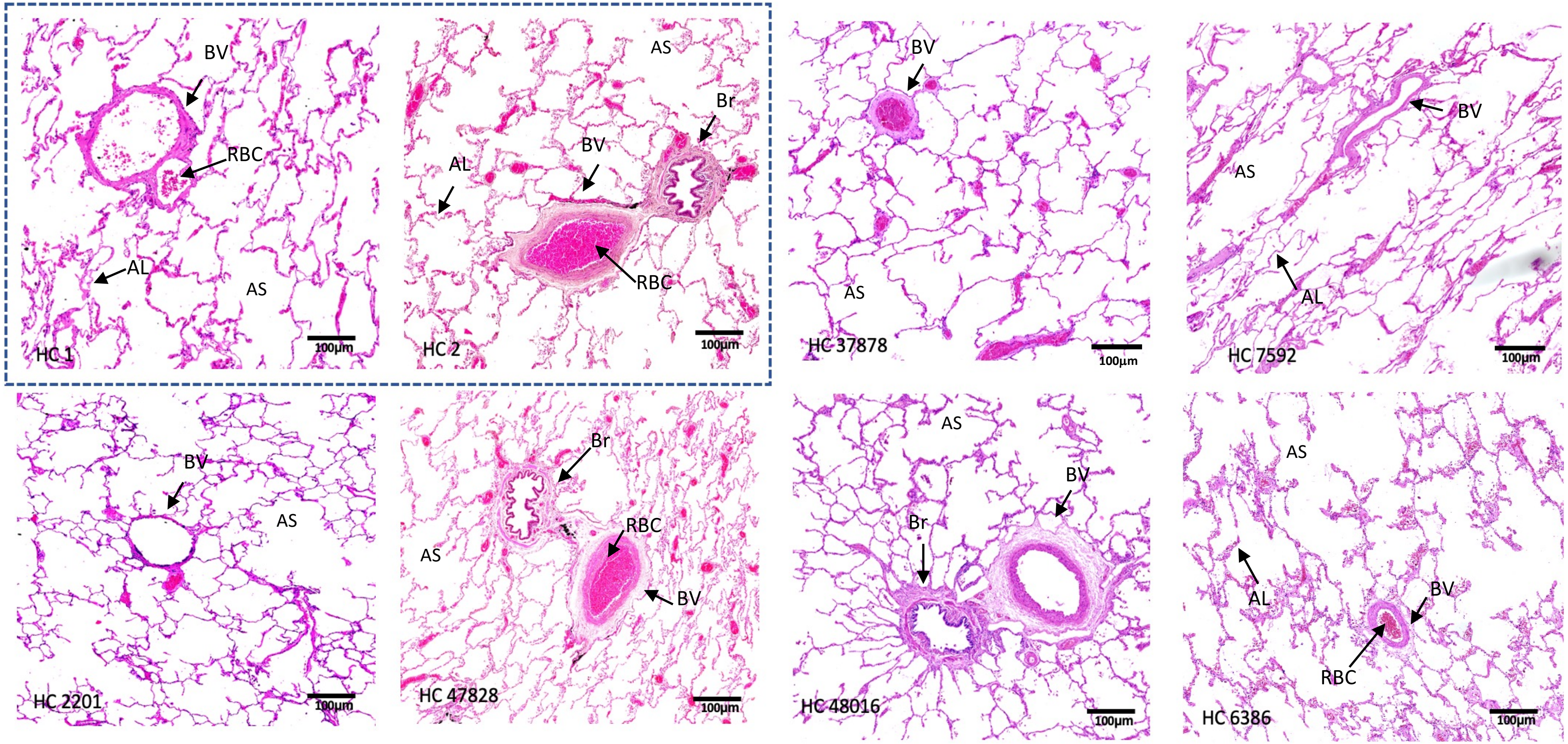

Supplementary Fig. 2. Representative H&E staining showing three histopathology states – Alveolitis, DAD and OP

A. 2x2mm regions of interest (ROI) sections from same lung samples used for IMC, 3 representative sections with alveolitis (ALV), diffuse alveolar damage (DAD) and organising pneumonia (OP) from 10 ALV; 8 DAD and 8 OP ROIs are shown in these figures; derived from n=12 patients.

B. Figure shows (in blue box), one of the two ROIs each for HC1 and HC2 which were submitted for IMC staining and spatial analyses. Outside blue box are ROIs from additional n=6 HC lungs for comparison (one representative ROI of 4 ROIs per individual lung sample is shown). All samples show typical thin unicellular alveolar lining (AL) with minimal number of immune cells. In some ROIs, normal broncho-vascular bundle (Br= bronchiole, BV = blood vessel) is evident. Varying sizes of normal blood vessels are seen (smaller – usually smaller pulmonary veins, larger with smooth muscle sheath are pulmonary veins or bronchial arteries).Alv -alveolitis, DAD - diffuse alveolar damage, OP - organising pneumonia. Overall, staining performed once, and on one day for samples from Figure (A) and once, on 2 different days for (B).

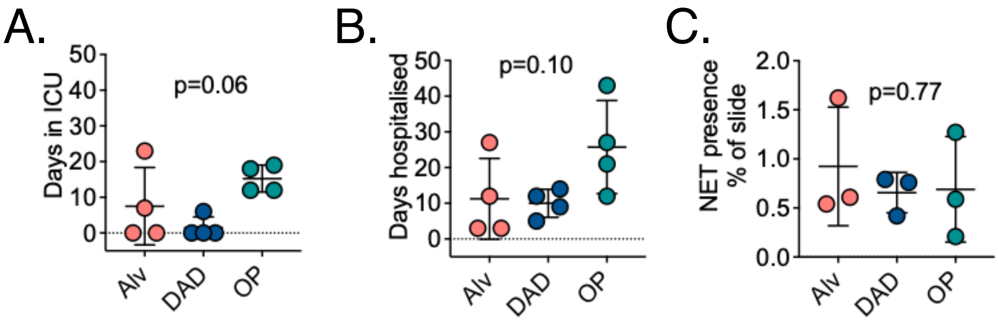

Supplementary Fig. 3. Correlation between clinical data and histopathology states

A. Graph showing total days in intensive care unit (ICU) up to the point of sampling and corresponding histopathology state for lung sections (n=12 patients) B. Graph showing number of days of hospitalization up to the point of sampling and corresponding histopathology state for lung sections. C. Graph showing the presence of NETs in slides (as a percentage of surface area; data from 20 with corresponding histopathology state). Normality tested with d'Agostino & Pearson test. Median and IQR shown, p value calculated using Kruskal-Wallis test with Dunn's multiple comparison test. Alv -alveolitis, DAD -diffuse alveolar damage, OP -organising pneumonia. NET - neutrophil extracellular traps. Source data are provided in the Source Data file.

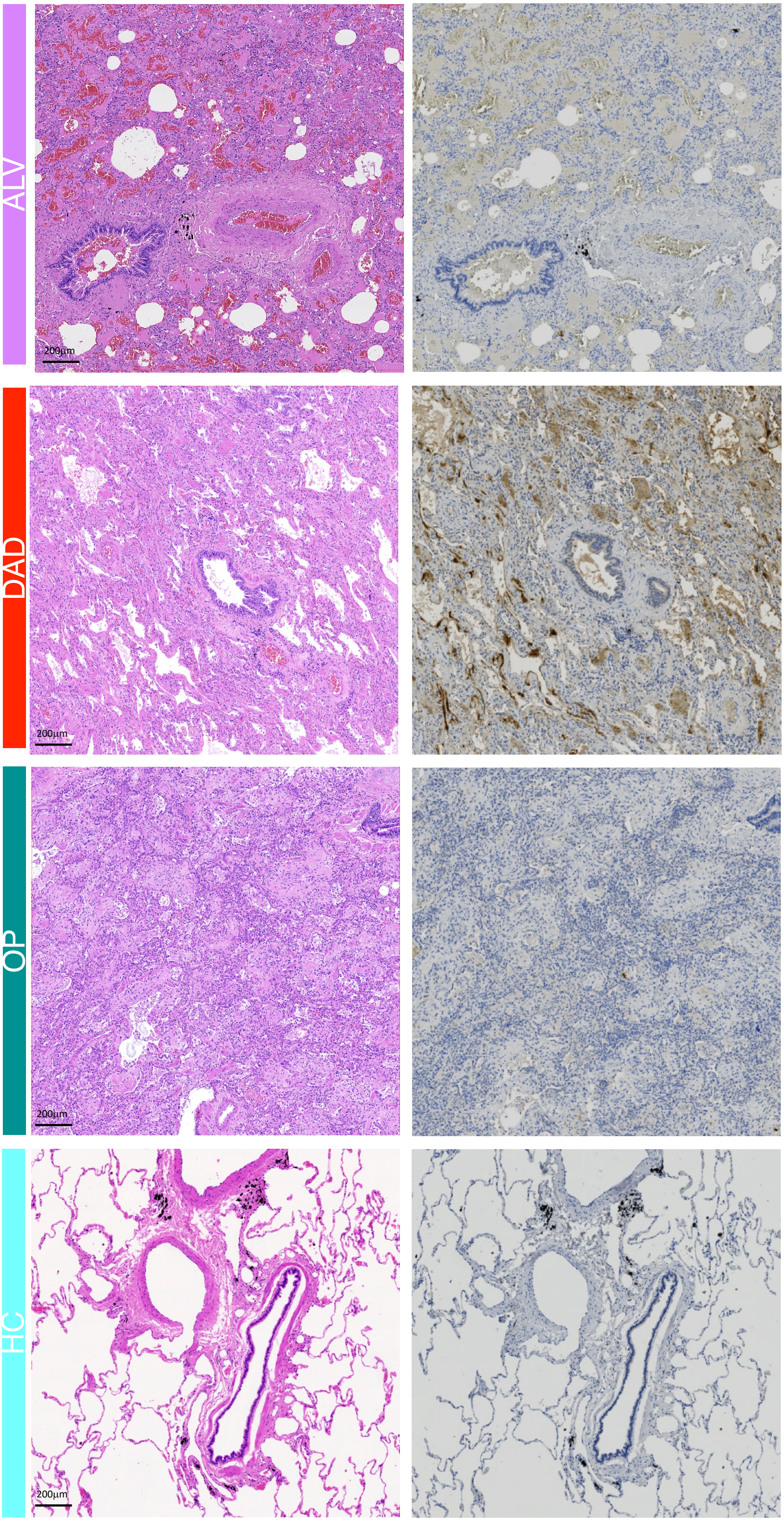

Supplementary Fig. 4

Supplementary Fig. 4. SARS-CoV-2 Nucleocapsid protein staining in lungs One representative ROI (of 3 available per lung shown) for SARS-CoV-2 Nucleocapsid protein staining by immunohistochemistry for lung sections with the histopathology states of alveolitis (ALV), diffuse alveolar damage (DAD) and organsing pneumonia (OP), and healthy control (HC). (One section shown of n= 10 ALV, n=8 DAD, n=8 OP and n=4 HC). Left column - H&E sections, right column - immunohistochemistry staining (brown) of nucleocapsid protein. One experiment performed for each sample.

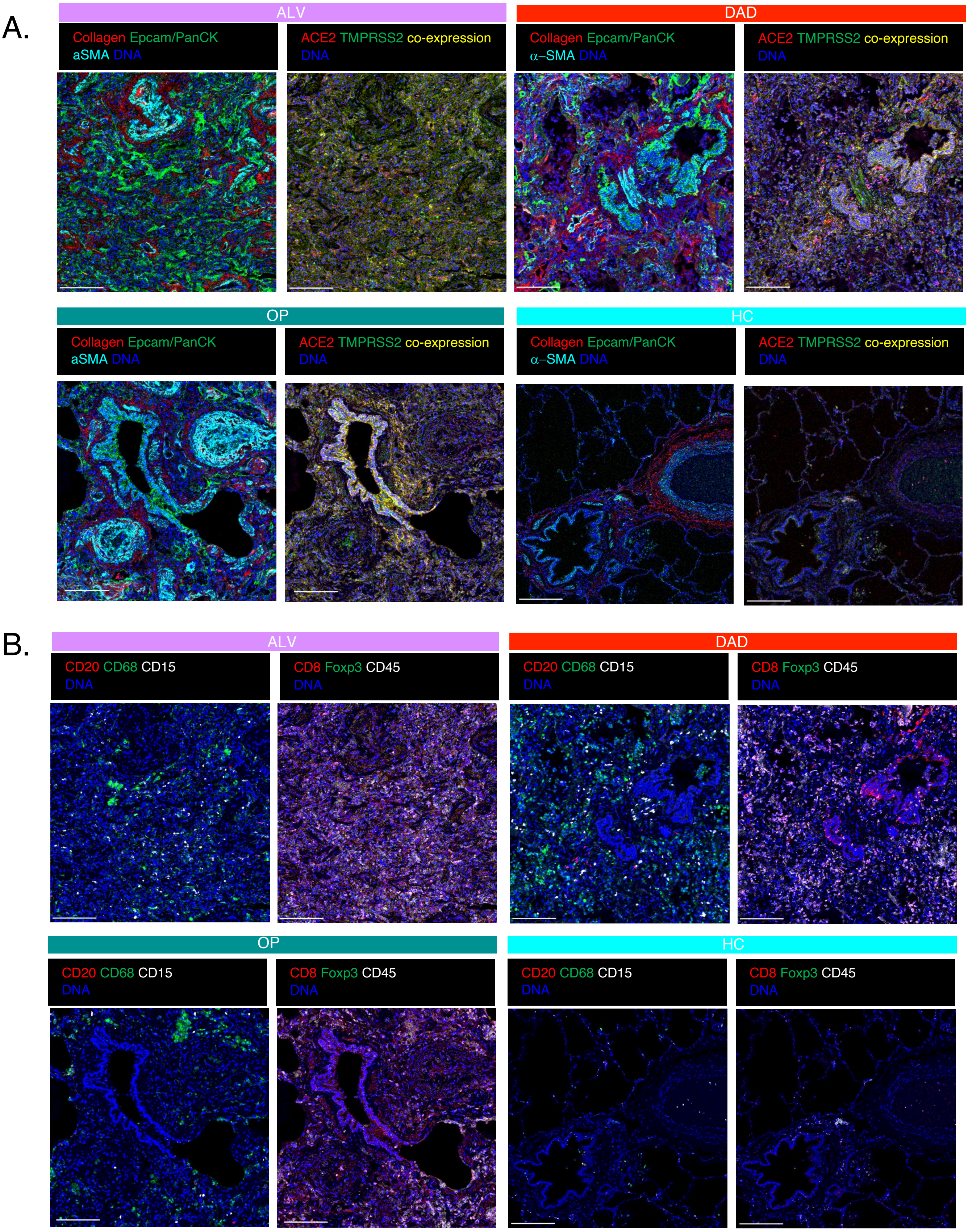

Supplementary Fig. 5

Supplementary Fig. 5. MCD images from imaging mass cytometry from the first 'sentinel' cohort experiment. n=3 patients (one ROI showing ALV, DAD and OP from each patient is shown, out of 3 ROIs per histopathology state, n=1 ALV, n=1 DAD and n=1 OP), and one of 3 ROIs from one healthy control lung

A. MCD images showing expression of structural cell markers (Col1a, EpCAM, PanCK, -SMA) and the viral integration receptors ACE2 and TMPRSS2. There was widespread ACE2 and TMPRSS2 expression in all histopathology states.
B. MCD images showing expression of key immune cell populations in same sections as (A); B cell (CD20); hematopoietic cells (CD45), CD8 T cells (CD8), regulatory T cells/ Tregs (FoxP3), macrophages (CD68). There were very low numbers of B cells and Tregs in lung sections from all three patients. All scale bars - 200 μm.

Histopathology states - Alv -alveolitis, DAD - diffuse alveolar damage, OP - organising pneumonia.

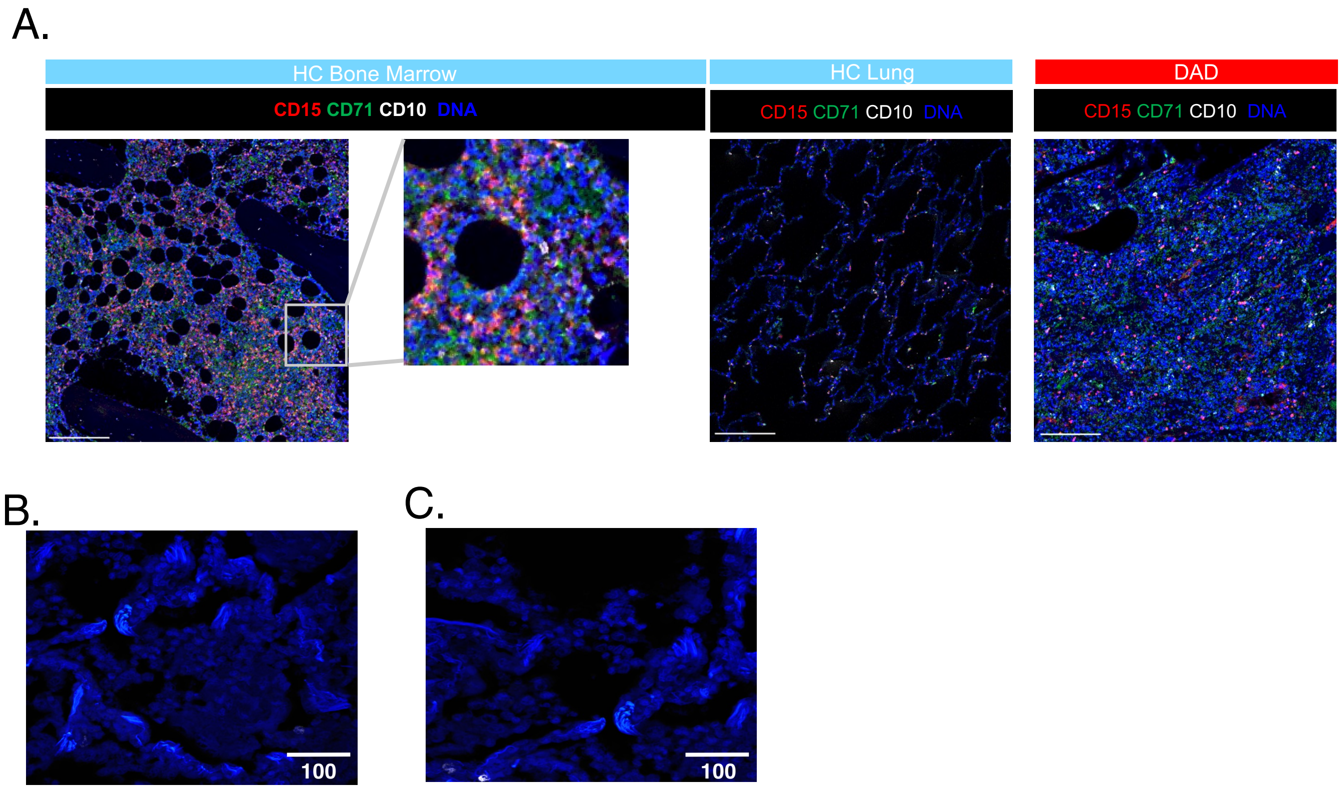

Supplementary Fig. 6 Selected controls for imaging mass cytometry (IMC) and immunofluorescence (IF) staining

A.MCD images show expression of CD10 and CD71 on CD15-expressing neutrophils in lung and bone marrow from a healthy control and a lung sample from a patient with DAD histopathology state (from 'sentinel cohort'). Scale bar 200 µm. One representative ROI of 3 ROIs is shown

B-C Isotype control for immunofluorescence staining for CD8/CD15 staining (Fig. 2D) and CD14/CD31/CD15 staining (Fig. 2E) performed on sections from same sample as Fig. 2D and C. One of 4 ROIs is shown. An isotype control is performed for every separate staining experiment.

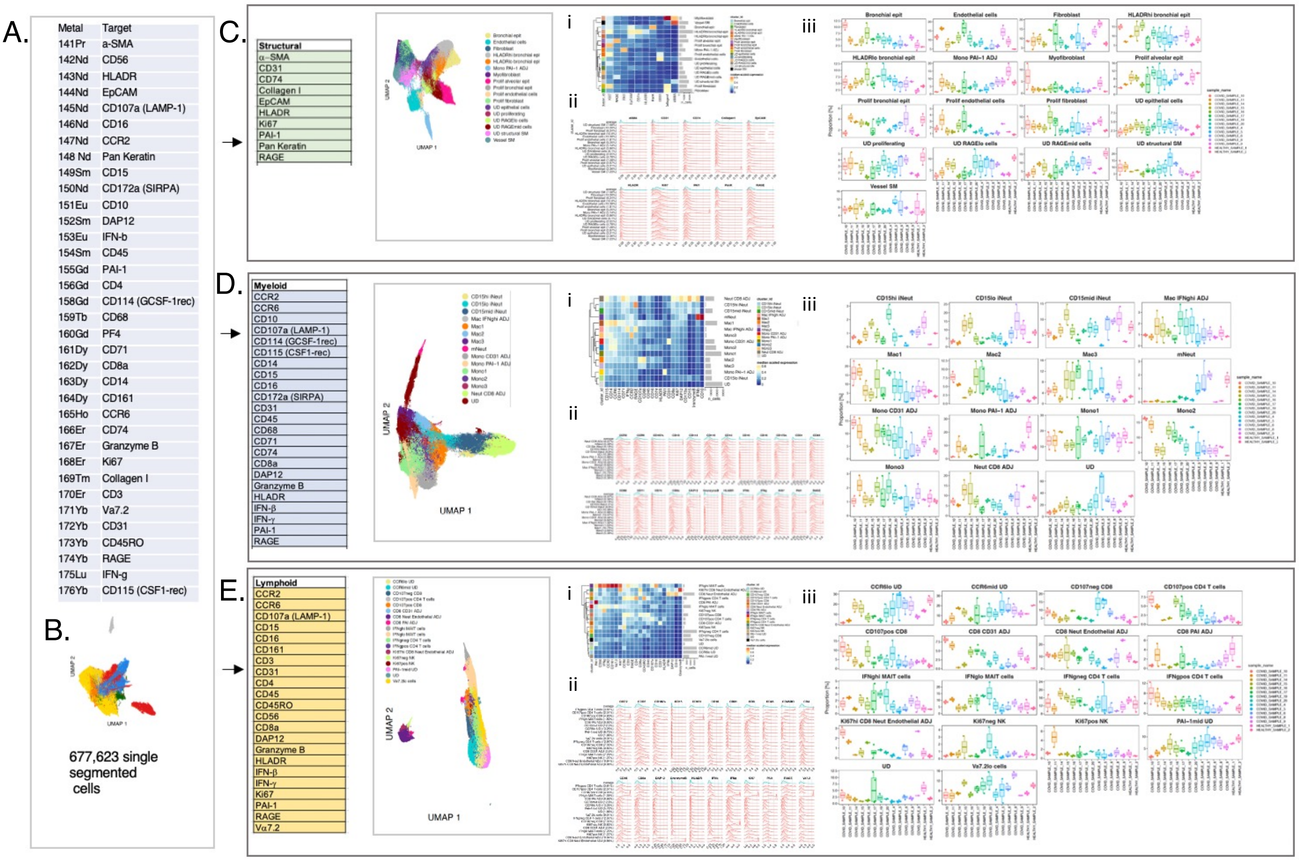

Supplementary Fig. 7 Cluster annotation workflow

A-E. Overall work flow for annotation of clusters obtained from single cell segmentation. A - 37-plex antibody panel and their respective metal tags. B - overall UMAP of clusters for n=677,623 unfiltered single cells from all patients (n=12) and healthy controls (n=2). Cells that did not show any antibody staining were then filtered from further analysis. The remaining cells (n=524,552) were grouped into three mega-clusters termed Structural, Myeloid and Lymphoid based on presence and/or absence of CD45, EPCAM, PanCK, CD31, α-SMA, CD56, Vα7.2, CD3, CD14, CD68, PF4 (for megakaryocyte) and CD15 expression. These three mega-clusters were then re-clustered and annotated according to heatmaps showing (i) median marker expression, (ii) expression density histograms which allowed better delineation of the range of marker expression, specifically differentiating low and negative expression levels and (iii) cluster distribution plots which showed the frequency of each cluster in different samples.

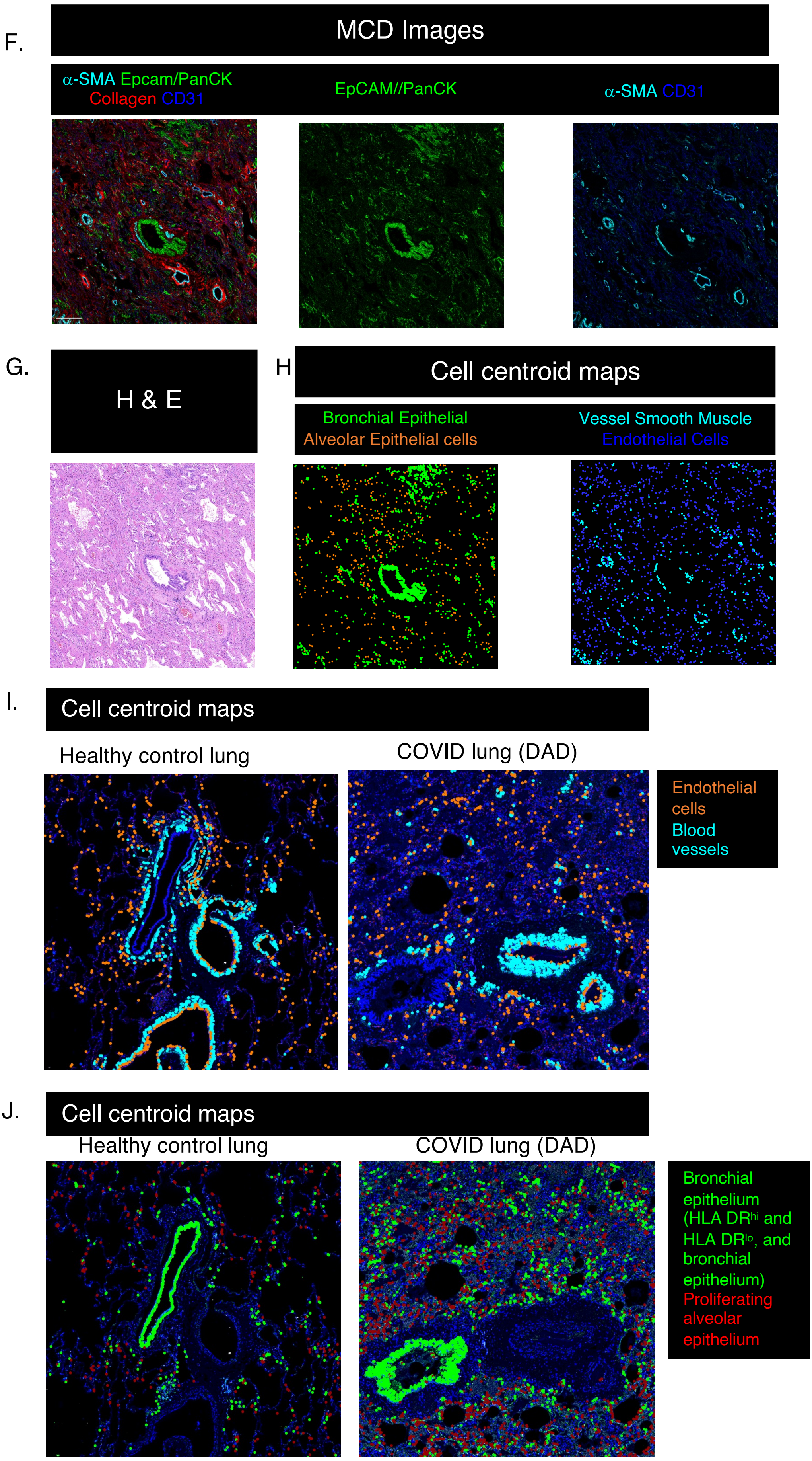
F.
MCD Images
α-SMA Epcam/PanCK
Collagen CD31
EpCAM//PanCK
α-SMA CD31
G.
H & E
H
Cell centroid maps
Bronchial Epithelial
Alveolar Epithelial cells
Vessel Smooth Muscle
Endothelial Cells
I.
Cell centroid maps
Healthy control lung
COVID lung (DAD)
Endothelial cells
Blood vessels
J.
Cell centroid maps
Healthy control lung
COVID lung (DAD)
Bronchial epithelium (HLA DR^{hi} and HLA DR^{lo}, and bronchial epithelium)
Proliferating alveolar epithelium

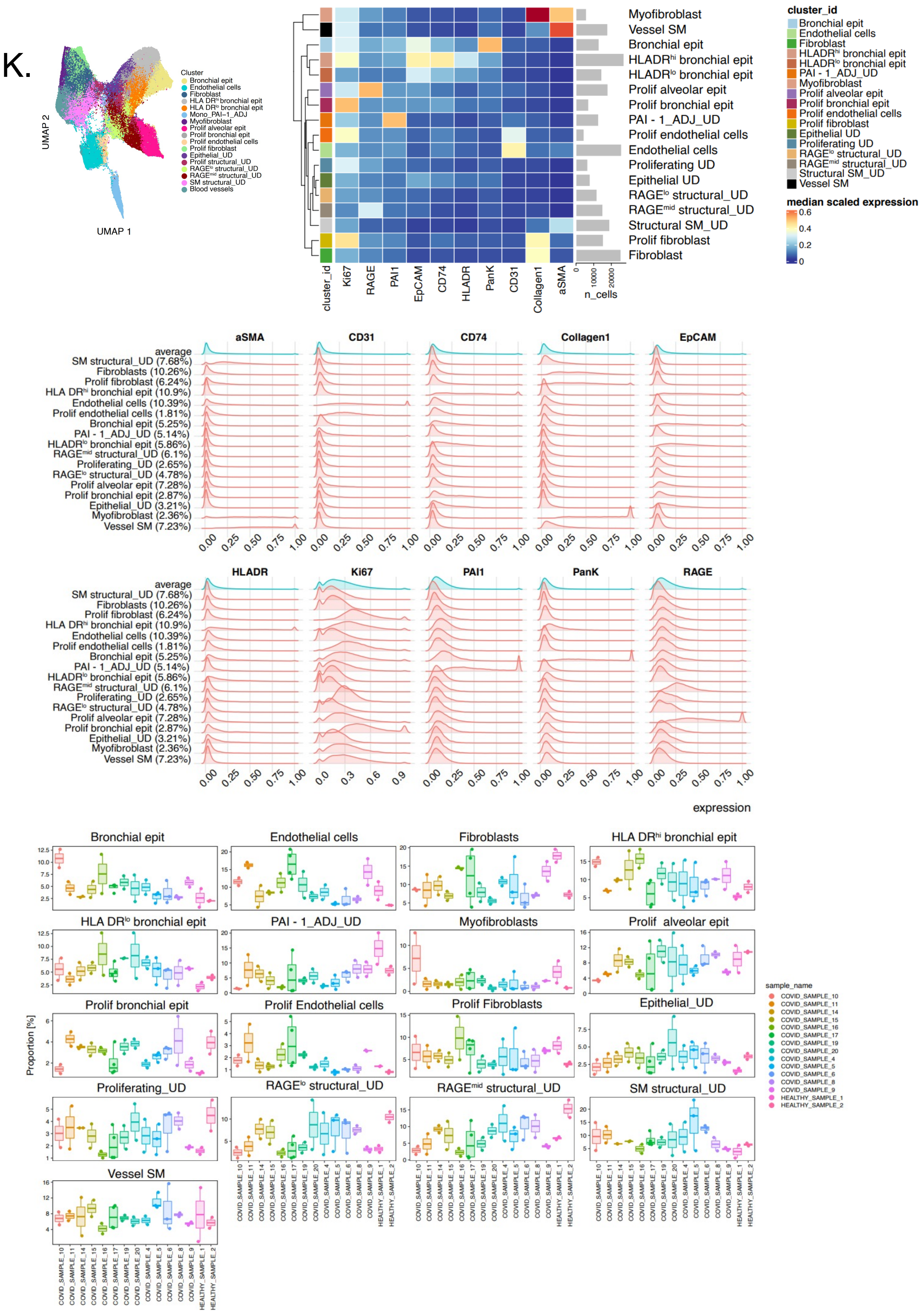
K.
UMAP 1
UMAP 2
Cluster
Bronchial epit
Endothelial cells
Fibroblast
HLA DR^hi bronchial epit
HLA DR^lo bronchial epit
Mono_PAI−1_ADJ
Myofibroblast
Prolif alveolar epit
Prolif bronchial epit
Prolif endothelial cells
Prolif fibroblast
Epithelial_UD
Prolif structural_UD
RAGE^lo structural_UD
RAGE^mid structural_UD
SM structural_UD
Blood vessels
Myofibroblast
Vessel SM
Bronchial epit
HLADR^hi bronchial epit
HLADR^lo bronchial epit
Prolif alveolar epit
Prolif bronchial epit
PAI - 1_ADJ_UD
Prolif endothelial cells
Endothelial cells
Proliferating UD
Epithelial UD
RAGE^lo structural_UD
RAGE^mid structural_UD
Structural SM_UD
Prolif fibroblast
Fibroblast
cluster_id
Ki67
RAGE
PAI1
EpCAM
CD74
HLADR
PanK
CD31
Collagen1
aSMA
n_cells
median scaled expression
aSMA
CD31
CD74
Collagen1
EpCAM
HLADR
Ki67
PAI1
PanK
RAGE
average
SM structural_UD (7.68%)
Fibroblasts (10.26%)
Prolif fibroblast (6.24%)
HLA DR^hi bronchial epit (10.9%)
Endothelial cells (10.39%)
Prolif endothelial cells (1.81%)
Bronchial epit (5.25%)
PAI - 1_ADJ_UD (5.14%)
HLADR^lo bronchial epit (5.86%)
RAGE^mid structural_UD (6.1%)
Proliferating_UD (2.65%)
RAGE^lo structural_UD (4.78%)
Prolif alveolar epit (7.28%)
Prolif bronchial epit (2.87%)
Epithelial_UD (3.21%)
Myofibroblast (2.36%)
Vessel SM (7.23%)
expression
Bronchial epit
Endothelial cells
Fibroblasts
HLA DR^hi bronchial epit
HLA DR^lo bronchial epit
PAI - 1_ADJ_UD
Myofibroblasts
Prolif alveolar epit
Prolif bronchial epit
Prolif Endothelial cells
Prolif Fibroblasts
Epithelial_UD
Proliferating_UD
RAGE^lo structural_UD
RAGE^mid structural_UD
SM structural_UD
Vessel SM
Proportion [%]
sample_name
COVID_SAMPLE_10
COVID_SAMPLE_11
COVID_SAMPLE_14
COVID_SAMPLE_15
COVID_SAMPLE_16
COVID_SAMPLE_17
COVID_SAMPLE_19
COVID_SAMPLE_20
COVID_SAMPLE_4
COVID_SAMPLE_5
COVID_SAMPLE_6
COVID_SAMPLE_8
COVID_SAMPLE_9
HEALTHY_SAMPLE_1
HEALTHY_SAMPLE_2

L.

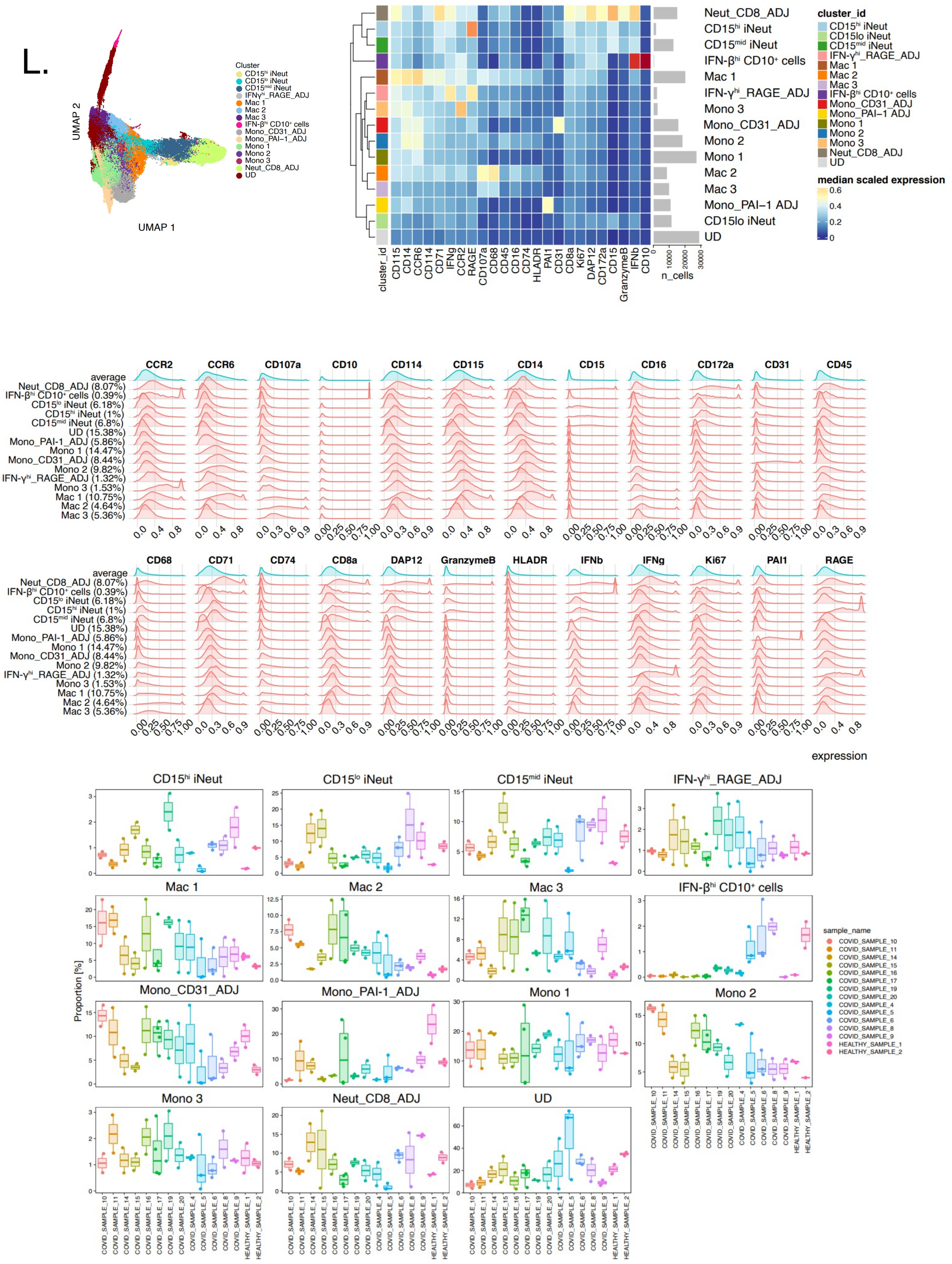

Cluster
CD15hi iNeut
CD15lo iNeut
CD15mid iNeut
IFNγhi_RAGE_ADJ
Mac 1
Mac 2
Mac 3
IFN-βhi CD10+ cells
Mono_CD31_ADJ
Mono_PAI-1_ADJ
Mono 1
Mono 2
Mono 3
Neut_CD8_ADJ
UD
UMAP 2
UMAP 1
cluster_id
CD115 CD14 CCR6 CD114 CD71 IFNg CCR2 RAGE CD107a CD68 CD45 CD16 CD74 HLADR PAI1 CD31 CD8a Ki67 DAP12 CD172a CD15 GranzymeB IFNb CD10
n_cells
median scaled expression
Neut_CD8_ADJ
CD15hi iNeut
CD15mid iNeut
IFN-βhi CD10+ cells
Mac 1
IFN-γhi_RAGE_ADJ
Mono 3
Mono_CD31_ADJ
Mono 2
Mono 1
Mac 2
Mac 3
Mono_PAI-1 ADJ
CD15lo iNeut
UD
average
Neut_CD8_ADJ (8.07%)
IFN-βhi CD10+ cells (0.39%)
CD15lo iNeut (6.18%)
CD15hi iNeut (1%)
CD15mid iNeut (6.8%)
UD (15.38%)
Mono_PAI-1_ADJ (5.86%)
Mono 1 (14.47%)
Mono_CD31_ADJ (8.44%)
Mono 2 (9.82%)
IFN-γhi_RAGE_ADJ (1.32%)
Mono 3 (1.53%)
Mac 1 (10.75%)
Mac 2 (4.64%)
Mac 3 (5.36%)
CCR2 CCR6 CD107a CD10 CD114 CD115 CD14 CD15 CD16 CD172a CD31 CD45
CD68 CD71 CD74 CD8a DAP12 GranzymeB HLADR IFNb IFNg Ki67 PAI1 RAGE
expression
Proportion [%]
CD15hi iNeut
CD15lo iNeut
CD15mid iNeut
IFN-γhi_RAGE_ADJ
Mac 1
Mac 2
Mac 3
IFN-βhi CD10+ cells
Mono_CD31_ADJ
Mono_PAI-1_ADJ
Mono 1
Mono 2
Mono 3
Neut_CD8_ADJ
UD
sample_name
COVID_SAMPLE_10
COVID_SAMPLE_11
COVID_SAMPLE_14
COVID_SAMPLE_15
COVID_SAMPLE_16
COVID_SAMPLE_17
COVID_SAMPLE_19
COVID_SAMPLE_20
COVID_SAMPLE_4
COVID_SAMPLE_5
COVID_SAMPLE_6
COVID_SAMPLE_8
COVID_SAMPLE_9
HEALTHY_SAMPLE_1
HEALTHY_SAMPLE_2

M.

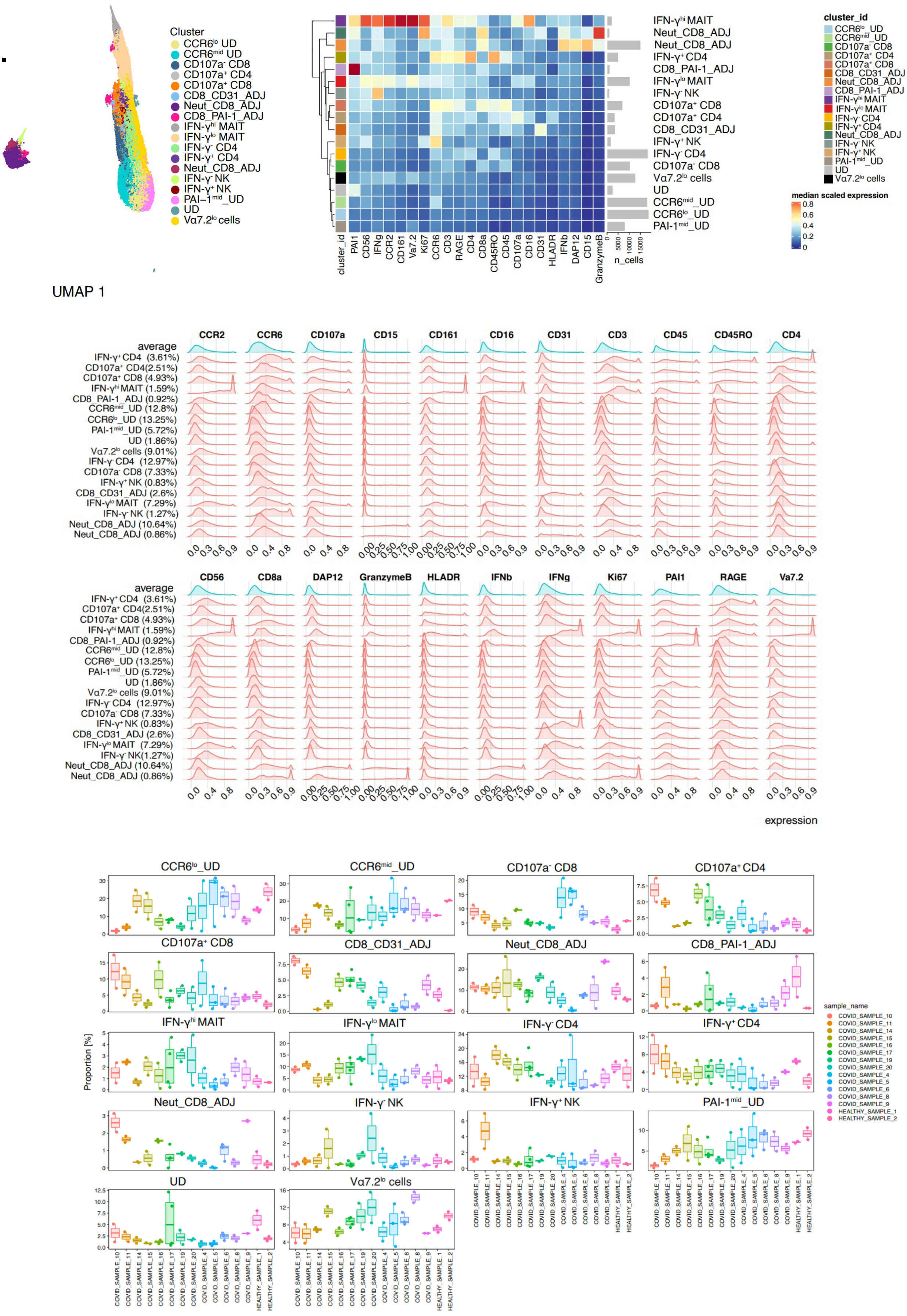

UMAP 2
UMAP 1
Cluster
CCR6lo UD
CCR6mid UD
CD107a- CD8
CD107a+ CD4
CD107a+ CD8
CD8_CD31_ADJ
Neut_CD8_ADJ
CD8_PAI-1_ADJ
IFN-γhi MAIT
IFN-γlo MAIT
IFN-γ- CD4
IFN-γ+ CD4
Neut_CD8_ADJ
IFN-γ- NK
IFN-γ+ NK
PAI-1mid_UD
UD
Vα7.2lo cells
cluster_id
n_cells
median scaled expression
PAI1
CD56
IFNg
CCR2
CD161
Vα7.2
Ki67
CCR6
CD3
RAGE
CD4
CD8a
CD45RO
CD45
CD107a
CD16
CD31
HLADR
IFNb
DAP12
CD15
GranzymeB
average
IFN-γ+ CD4 (3.61%)
CD107a+ CD4(2.51%)
CD107a+ CD8 (4.93%)
IFN-γhi MAIT (1.59%)
CD8_PAI-1_ADJ (0.92%)
CCR6mid_UD (12.8%)
CCR6lo_UD (13.25%)
PAI-1mid_UD (5.72%)
UD (1.86%)
Vα7.2lo cells (9.01%)
IFN-γ- CD4 (12.97%)
CD107a- CD8 (7.33%)
IFN-γ+ NK (0.83%)
CD8_CD31_ADJ (2.6%)
IFN-γlo MAIT (7.29%)
IFN-γ- NK (1.27%)
Neut_CD8_ADJ (10.64%)
Neut_CD8_ADJ (0.86%)
expression
Proportion [%]
sample_name
COVID_SAMPLE_10
COVID_SAMPLE_11
COVID_SAMPLE_14
COVID_SAMPLE_15
COVID_SAMPLE_16
COVID_SAMPLE_17
COVID_SAMPLE_19
COVID_SAMPLE_20
COVID_SAMPLE_4
COVID_SAMPLE_5
COVID_SAMPLE_6
COVID_SAMPLE_8
COVID_SAMPLE_9
HEALTHY_SAMPLE_1
HEALTHY_SAMPLE_2

N.

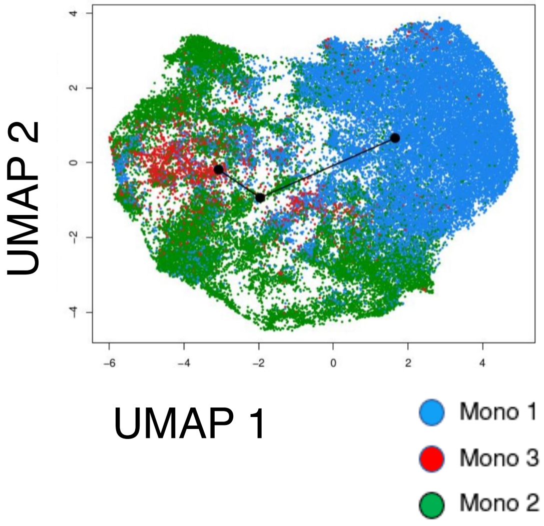

O.

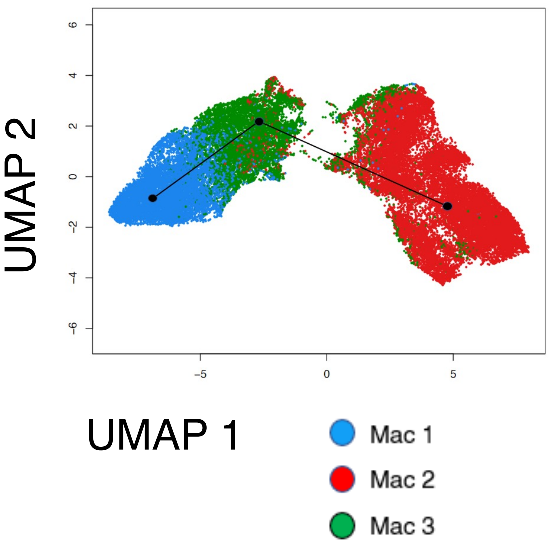

P.

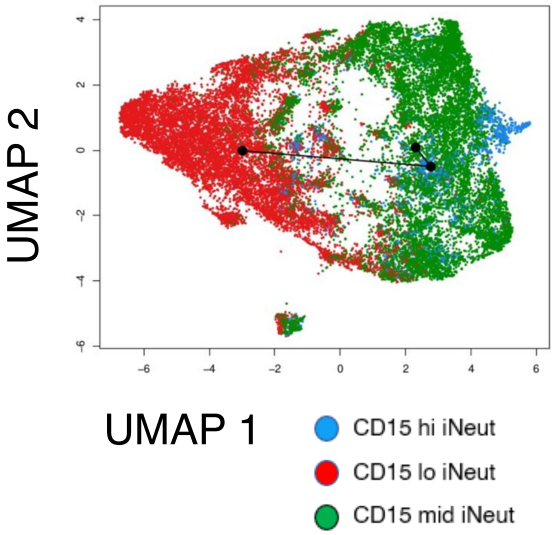

Supplementary Fig. 7

F-J. To further define cluster identities, the spatial location of clusters was visualised using cell centroid plots and mapped onto an adjacent H&E slide (F-J). An exemplar series of selected markers from MCD images of a sample with DAD (F), its corresponding H&E section (G) and the cell centroid map showing the corresponding final identity of the cells (H) is shown. I-J - examples of final identity of annotated cells – endothelial cells, blood vessels and proliferating endothelial cells in healthy lungs and DAD (I) and bronchial epithelium and proliferating alveolar epithelium (J).

K-M. High resolution and larger figure for figures (C-E).

N-P. Final annotations were also aided by Pseudotime analysis of selected immune cells where cell differentiation trajectories were expected e.g. monocytes are expected to differentiate to macrophages thus early monocytes (less differentiated monocytes to more differentiated monocytes or 'monocyte-macrophage' (N); and from less mature to more mature macrophages (O) and from very immature neutrophils to less immature neutrophils (P). No directionality is inferred in these Pseudotime analyses, rather connectivity from one to another.

Source data for the graphs are provided in the Source Data file. For the graphs, boxes represent median and IQR. No statistical analyses were performed for the graphs as these were used as visual guides to identify unusual pattern of cell abundance. All graphs and data were for n=12 patients; ALV (n=10 ROIs), DAD (n=8 ROIs), OP (n=8 ROIs). Pseudotime analysis performed with Slingshot.

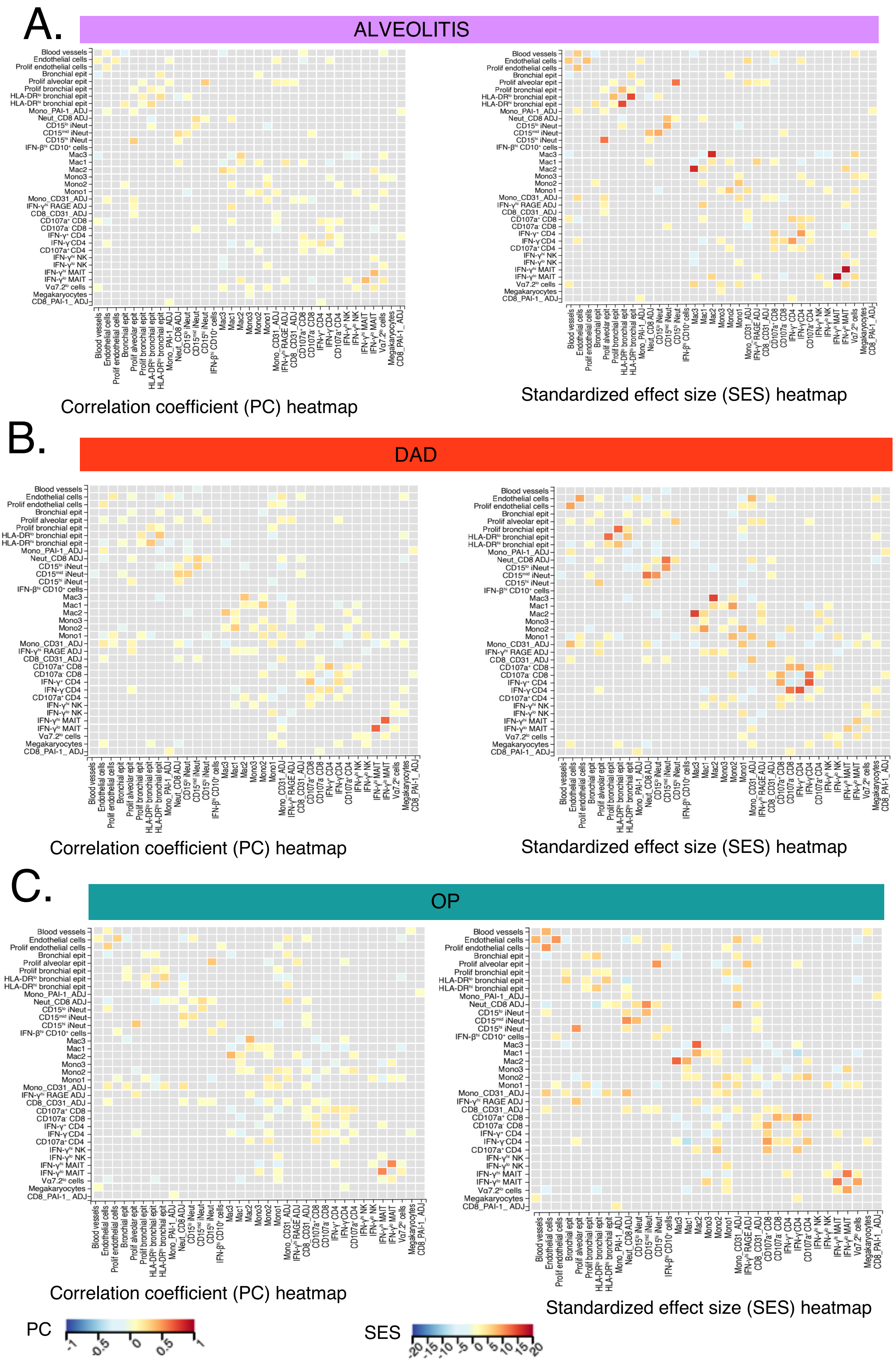
A.
ALVEOLITIS
Correlation coefficient (PC) heatmap
Standardized effect size (SES) heatmap
B.
DAD
Correlation coefficient (PC) heatmap
Standardized effect size (SES) heatmap
C.
OP
Correlation coefficient (PC) heatmap
Standardized effect size (SES) heatmap
Blood vessels
Endothelial cells
Prolif endothelial cells
Bronchial epit
Prolif alveolar epit
Prolif bronchial epit
HLA-DRlo bronchial epit
HLA-DRhi bronchial epit
Mono_PAI-1_ADJ
Neut_CD8 ADJ
CD15lo iNeut
CD15mid iNeut
CD15hi iNeut
IFN-βhi CD10+ cells
Mac3
Mac1
Mac2
Mono3
Mono2
Mono1
Mono_CD31_ADJ
IFN-γhi RAGE ADJ
CD8_CD31_ADJ
CD107a+ CD8
CD107a- CD8
IFN-γ+ CD4
IFN-γ-CD4
CD107a+ CD4
IFN-γhi NK
IFN-γlo NK
IFN-γhi MAIT
IFN-γlo MAIT
Vα7.2lo cells
Megakaryocytes
CD8_PAI-1_ADJ
PC
-1 -0.5 0 0.5 1
SES
-20 -15 -10 -5 0 5 10 15 20

Supplementary Fig. 8 Output from quadrat correlation matrix (QCM) analysis
A-C. Heat map showing correlation coefficient derived using partial correlation (PC) methods and standardised effect size of correlation for pairs that are statistically significantly correlated (FDR $q<0.05$) after adjusting for multiple comparisons. n=479,349 single cell (excluding those with no markers and undefined clusters) from n=10 ROIs for ALV, n=8 ROIs for DAD and n=8 ROIs for OP; n=12 patients in total. All performed in one batch (i.e. one experiment). For all histopathology states, pairs with borderline FDR q values (i.e. 0.05 to 0.10) were also examined as agreed by the consortium pre-analysis.

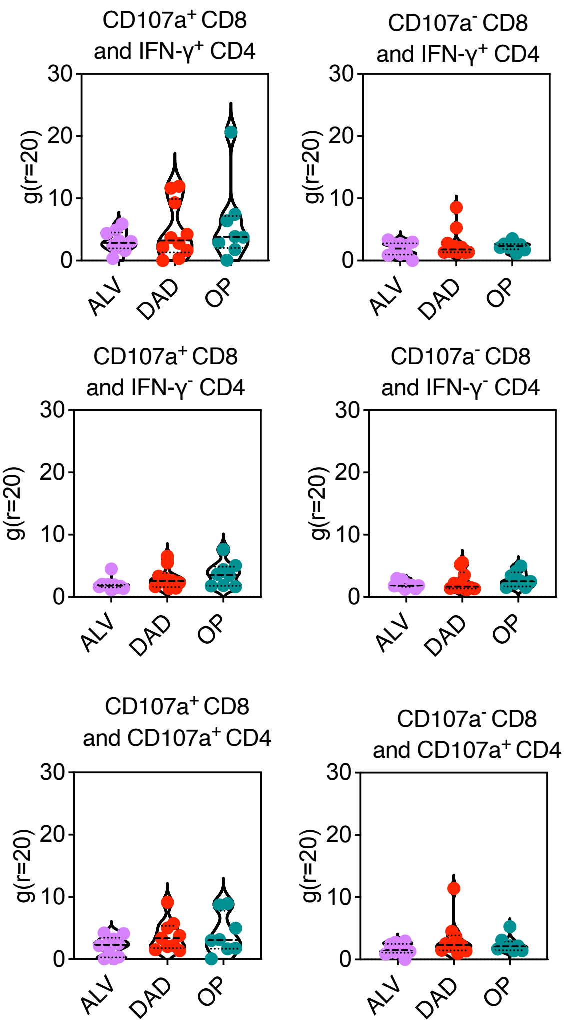

Supplementary Fig. 9. Cross- PCF output [g(r=20)] for selected pairs of CD4 and CD8 T cell subsets.

Graphs show g(r=20) values for CD8 and CD4 T cell subset pairs in ROIs from all patients (n=12) placed into histopathology states (ALV, DAD and OP); ALV (n=10 ROIs), DAD (n=8 ROIs), OP (n=8 ROIs). g(r=20) value greater than 2 implies co-location between the two cell types. Source data are provided in the Source Data file. Median values are shown with IQR.

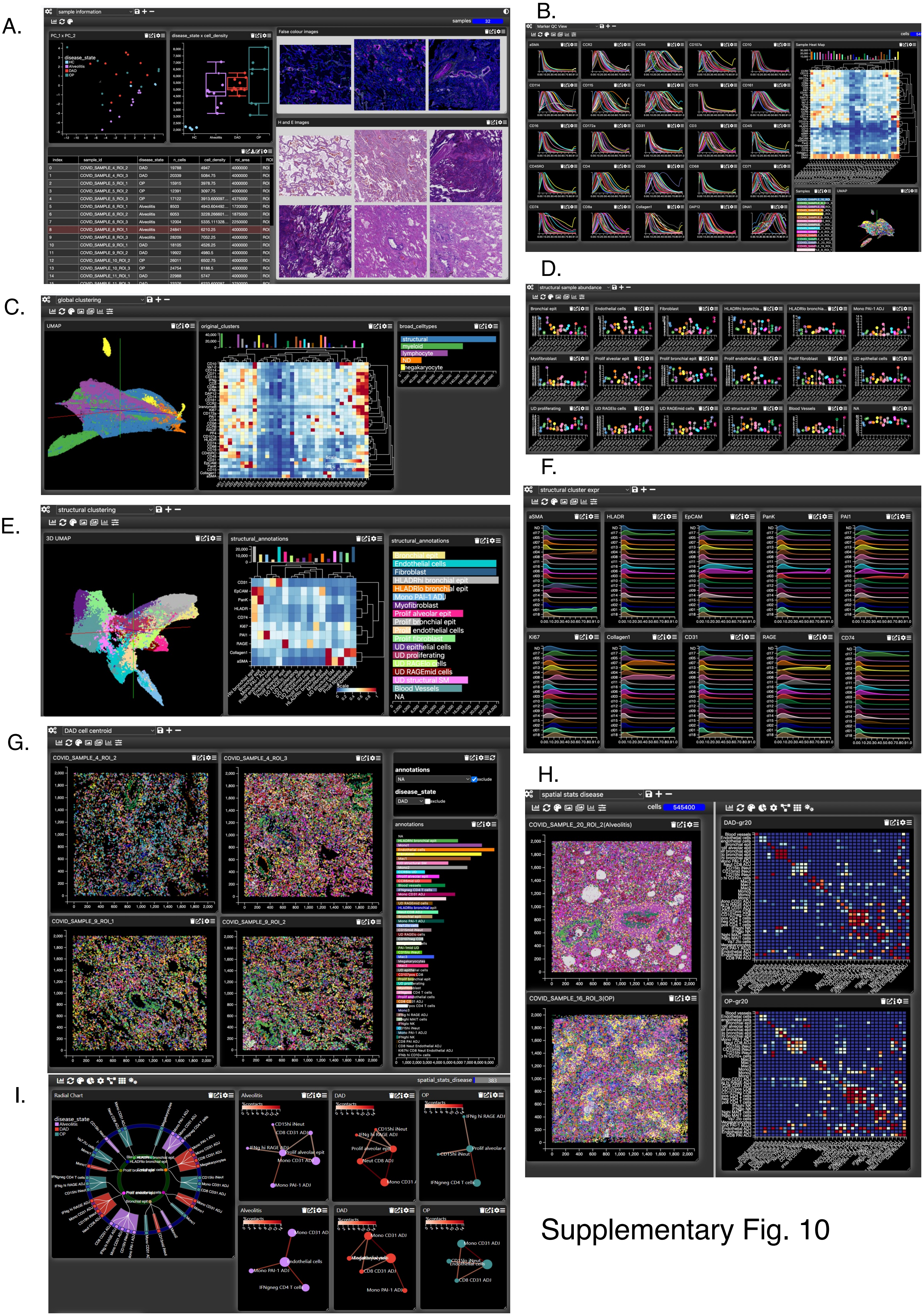

Supplementary Fig. 10

Supplementary Fig. 10. MCD software

Screenshots demonstrating the functionality of Multi-Dimensional Viewer (MDV), which is introduced as a public resource and platform in visualization and analysis of imaging mass cytometry data. Following data upload to MDV it provides a drop-down menu of several interactive views encompassing quality control metrics, clustering, visualization of cell centroid maps and relevant spatial statistics. A video walkthrough of the functionality exemplar of MDV is provided (https://drive.google.com/file/d/1E0n5iwuexNGTCFVw8UgUR5_rI63AZEYa/view).

A. Sample information view with principal component analyses of included samples, cell numbers per sample and disease state, information on regions of interest included in the analysis including surface are ablated, false colour images and representative H and E images

B. Quality control view showing median marker staining intensity per sample. Data is visualized on histograms, heatmaps showing the median marker expression per sample as well as UMAP displays coloured by sample

C. Global clustering view showing high level cluster information visualized with UMAP, heatmaps showing median expression density, and annotation into structural, myeloid, lymphocyte and megakaryocyte sub clusters.

D, E, F. Representative example of visualizations for sub- clustering of structural cells. D. annotated cluster distribution plots showing abundance of each cluster per sample, E. UMAP of structural cell clusters, with accompanying annotations and heatmap showing median marker expression. F. Scaled marker expression density plots which show the range of marker expression per defined cluster. All these views are interactive, enabling the user to closely examine the features of a cell cluster/clusters of interest.

G. Cell centroid representations of annotated cell clusters with regions of interest in the histopathological state diffuse alveolar damage (DAD) as an exemplar. This view allows the user to examine the spatial distribution of selected clusters for further robustness and accuracy of annotation as well as obtain a qualitative view of cell type co – location

H. Spatial analysis views showing the g(r=20) heatmaps for 2 histopathological states (DAD and OP) for identification of co – locating cell pairs. Interactive representation of this co – location can be obtained by mapping of the cell pair spatial location on to representative H&E images on the left panel

I. Exemplar spatial analysis views showing spatial connectivity radial map for structural cells and spatial connectivity diagrams for proliferating alveolar epithelial cells and endothelial cells in the different histopathological states

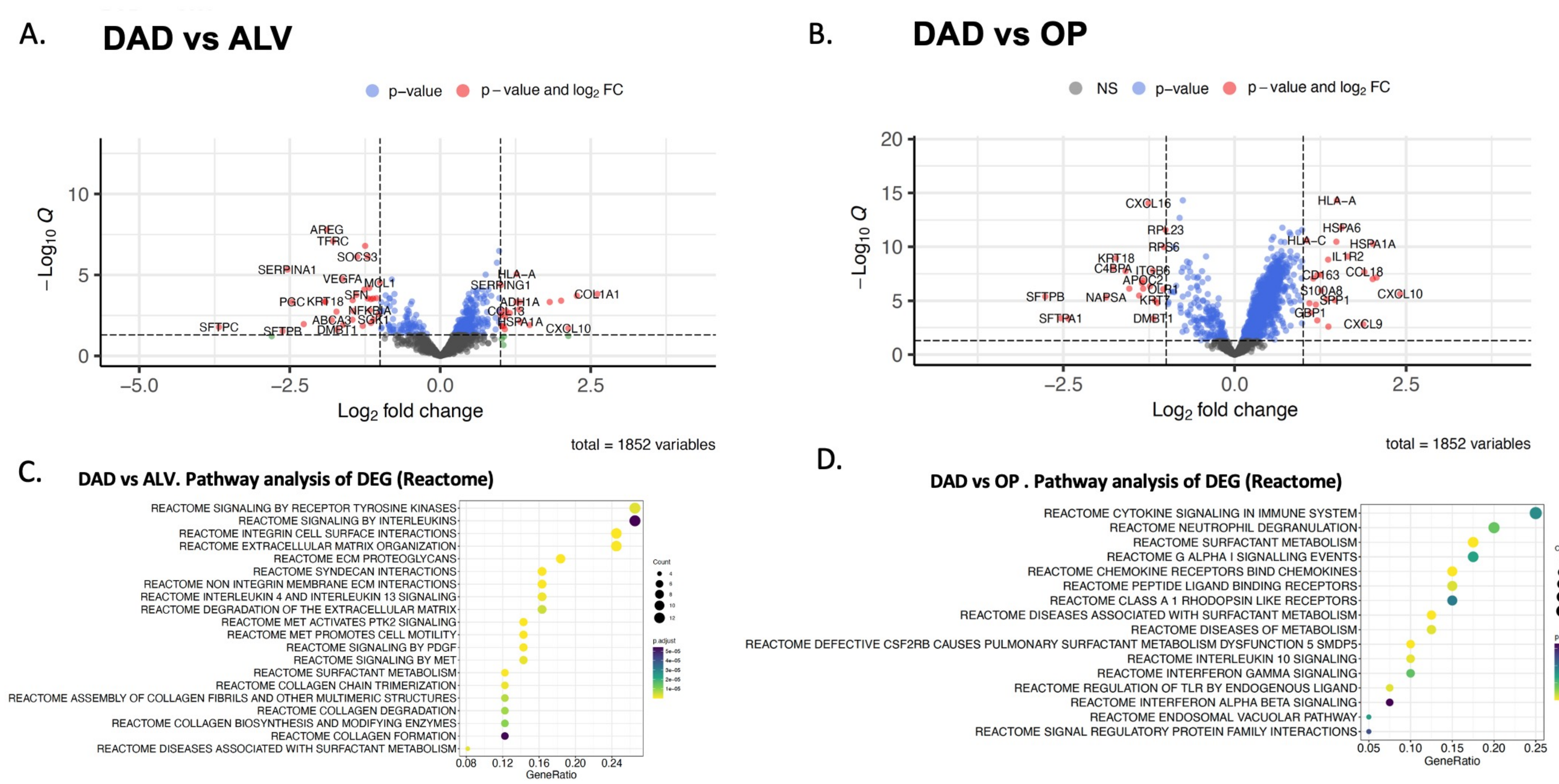
A.
DAD vs ALV
p–value
p – value and log2 FC
AREG
TFRC
SOCS3
SERPINA1
VEGFA
MCL1
SFN
PGC
KRT18
NFKBIA
ABCA3
SGK1
SFTPC
SFTPB
DMBT1
HLA–A
SERPING1
ADH1A
CCL13
HSPA1A
CXCL10
COL1A1
– Log10 Q
Log2 fold change
total = 1852 variables
B.
DAD vs OP
NS
p–value
p – value and log2 FC
CXCL16
RPL23
RPS6
KRT18
C4BPA
ITGB6
APOC2
OLR1
KRT7
NAPSA
SFTPB
SFTPA1
DMBT1
HLA–A
HSPA6
HLA–C
HSPA1A
IL1R2
CD163
CCL18
S100A8
SPP1
CXCL10
GBP1
CXCL9
– Log10 Q
Log2 fold change
total = 1852 variables
C.
DAD vs ALV. Pathway analysis of DEG (Reactome)
REACTOME SIGNALING BY RECEPTOR TYROSINE KINASES
REACTOME SIGNALING BY INTERLEUKINS
REACTOME INTEGRIN CELL SURFACE INTERACTIONS
REACTOME EXTRACELLULAR MATRIX ORGANIZATION
REACTOME ECM PROTEOGLYCANS
REACTOME SYNDECAN INTERACTIONS
REACTOME NON INTEGRIN MEMBRANE ECM INTERACTIONS
REACTOME INTERLEUKIN 4 AND INTERLEUKIN 13 SIGNALING
REACTOME DEGRADATION OF THE EXTRACELLULAR MATRIX
REACTOME MET ACTIVATES PTK2 SIGNALING
REACTOME MET PROMOTES CELL MOTILITY
REACTOME SIGNALING BY PDGF
REACTOME SIGNALING BY MET
REACTOME SURFACTANT METABOLISM
REACTOME COLLAGEN CHAIN TRIMERIZATION
REACTOME ASSEMBLY OF COLLAGEN FIBRILS AND OTHER MULTIMERIC STRUCTURES
REACTOME COLLAGEN DEGRADATION
REACTOME COLLAGEN BIOSYNTHESIS AND MODIFYING ENZYMES
REACTOME COLLAGEN FORMATION
REACTOME DISEASES ASSOCIATED WITH SURFACTANT METABOLISM
GeneRatio
Count
p.adjust
D.
DAD vs OP . Pathway analysis of DEG (Reactome)
REACTOME CYTOKINE SIGNALING IN IMMUNE SYSTEM
REACTOME NEUTROPHIL DEGRANULATION
REACTOME SURFACTANT METABOLISM
REACTOME G ALPHA I SIGNALLING EVENTS
REACTOME CHEMOKINE RECEPTORS BIND CHEMOKINES
REACTOME PEPTIDE LIGAND BINDING RECEPTORS
REACTOME CLASS A 1 RHODOPSIN LIKE RECEPTORS
REACTOME DISEASES ASSOCIATED WITH SURFACTANT METABOLISM
REACTOME DISEASES OF METABOLISM
REACTOME DEFECTIVE CSF2RB CAUSES PULMONARY SURFACTANT METABOLISM DYSFUNCTION 5 SMDP5
REACTOME INTERLEUKIN 10 SIGNALING
REACTOME INTERFERON GAMMA SIGNALING
REACTOME REGULATION OF TLR BY ENDOGENOUS LIGAND
REACTOME INTERFERON ALPHA BETA SIGNALING
REACTOME ENDOSOMAL VACUOLAR PATHWAY
REACTOME SIGNAL REGULATORY PROTEIN FAMILY INTERACTIONS
GeneRatio
Count
p.adjust

Supplementary Fig. 11. Analysis of gene expression profile of specific areas from three patients with COVID.
Gene expression profiles of cells (mean – 988 cells) in n=46 AOIs from n=3 COVID patients (published in Cross A.R. et al 2022)[52] were re-organised according to ALV, DAD and OP histopathology states and re-analysed. A-B. DEGs derived using Enhanced Volcano package comparing DAD with ALV, and DAD with OP (B). C-D. Pathway analysis with Reactome showed interleukin and tyrosine kinase signalling pathways as those with top gene ratios in DAD vs ALV (C) and cytokine signalling and neutrophil degranulation in DAD v OP (D). Both these data support excess neutrophil-related activity in ALV compared to the other histopathology states, enhancing our current data but does not conclude on presence and location of neutrophils.

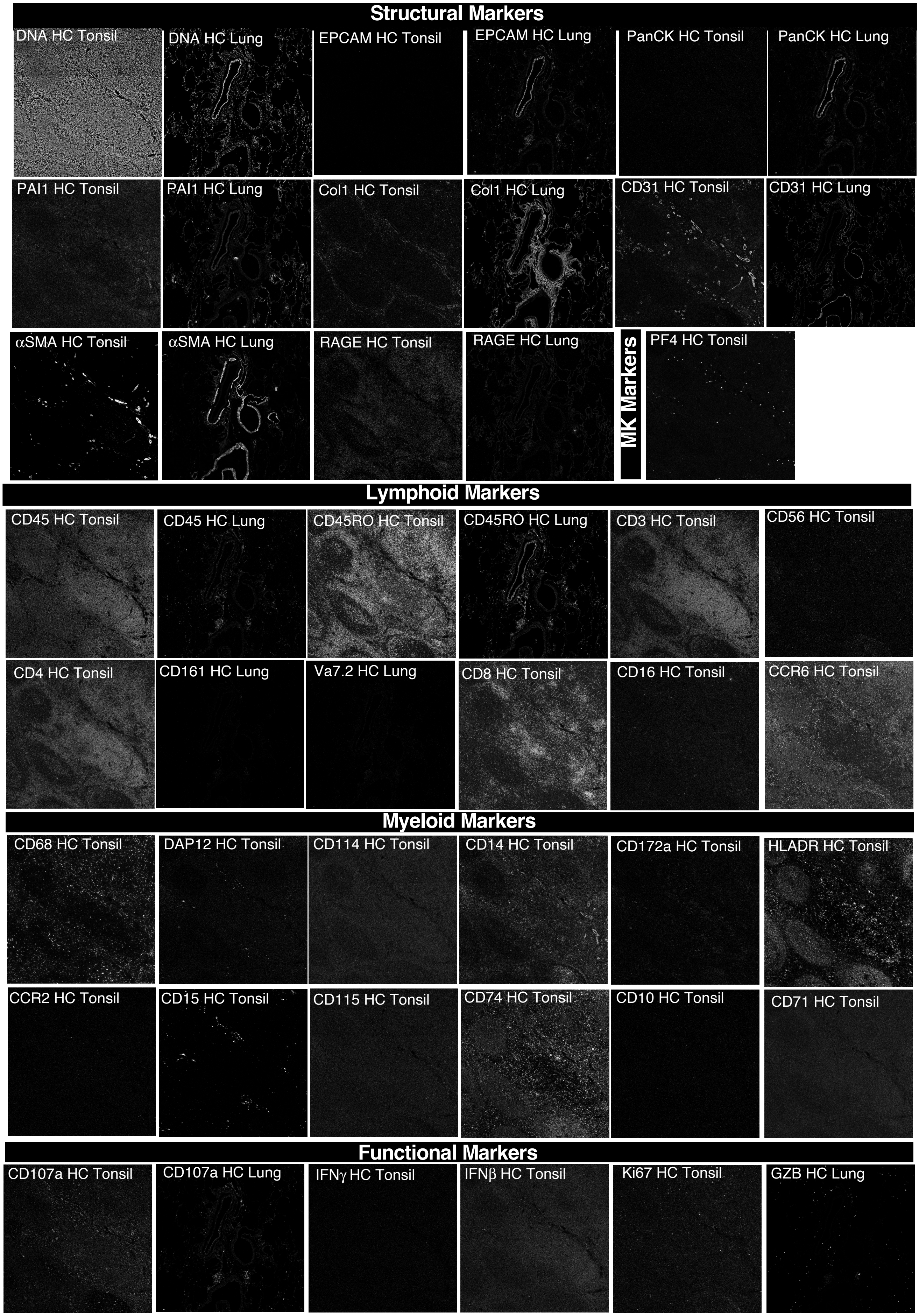
Structural Markers
DNA HC Tonsil
DNA HC Lung
EPCAM HC Tonsil
EPCAM HC Lung
PanCK HC Tonsil
PanCK HC Lung
PAI1 HC Tonsil
PAI1 HC Lung
Col1 HC Tonsil
Col1 HC Lung
CD31 HC Tonsil
CD31 HC Lung
αSMA HC Tonsil
αSMA HC Lung
RAGE HC Tonsil
RAGE HC Lung
MK Markers
PF4 HC Tonsil
Lymphoid Markers
CD45 HC Tonsil
CD45 HC Lung
CD45RO HC Tonsil
CD45RO HC Lung
CD3 HC Tonsil
CD56 HC Tonsil
CD4 HC Tonsil
CD161 HC Lung
Va7.2 HC Lung
CD8 HC Tonsil
CD16 HC Tonsil
CCR6 HC Tonsil
Myeloid Markers
CD68 HC Tonsil
DAP12 HC Tonsil
CD114 HC Tonsil
CD14 HC Tonsil
CD172a HC Tonsil
HLADR HC Tonsil
CCR2 HC Tonsil
CD15 HC Tonsil
CD115 HC Tonsil
CD74 HC Tonsil
CD10 HC Tonsil
CD71 HC Tonsil
Functional Markers
CD107a HC Tonsil
CD107a HC Lung
IFNγ HC Tonsil
IFNβ HC Tonsil
Ki67 HC Tonsil
GZB HC Lung

Supplementary Fig. 12 . Representative raw IMC staining shown for immune and structural markers in healthy control tonsil (n=1 individuals, n=2 ROIs) and lung (n=12 patient, n=26 ROIs). ROI size - 2mmx2mm. MK – megakaryocyte, HC – healthy control.

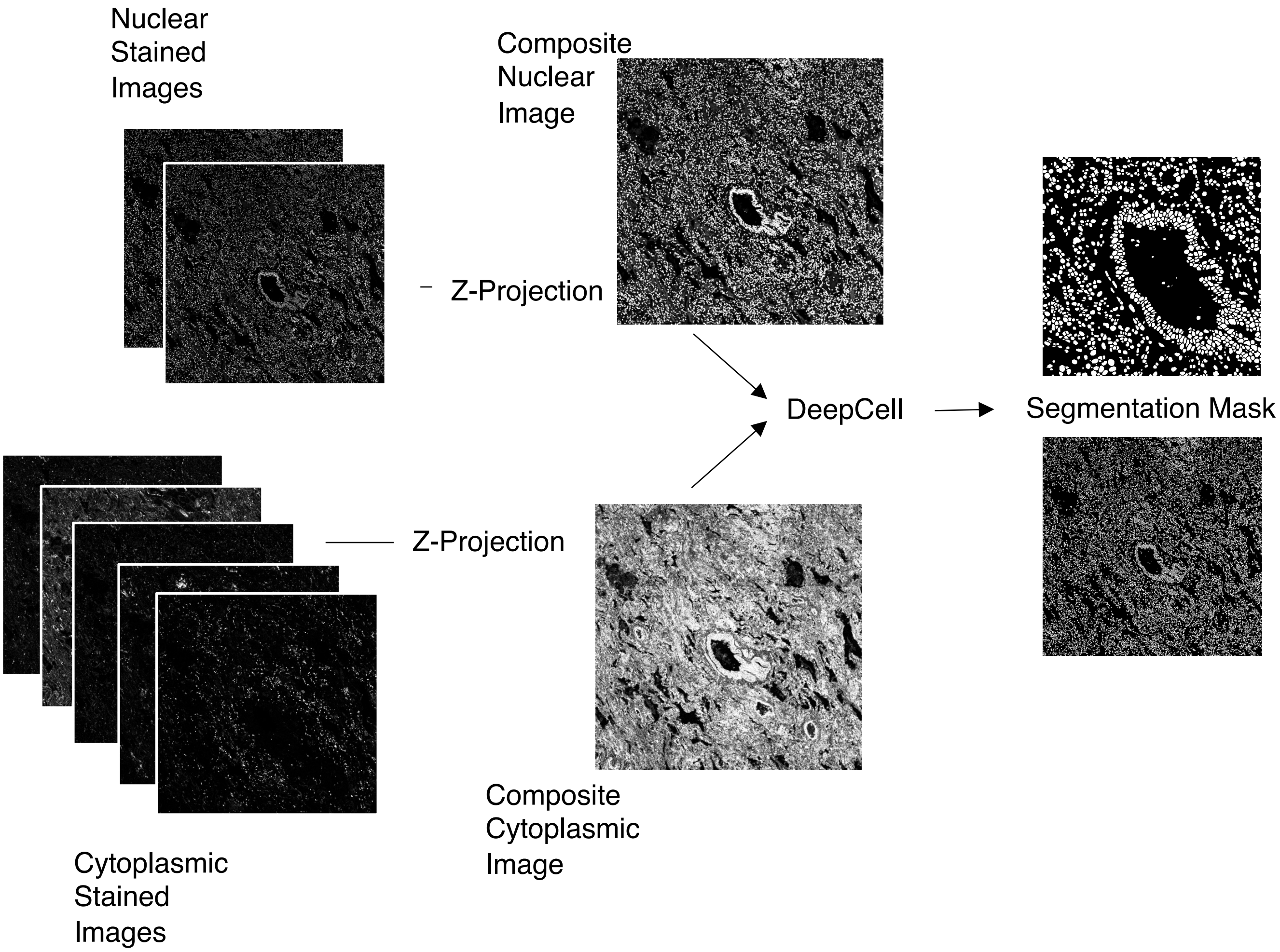

Supplementary Fig. 13. Single cell segmentation workflow using DeepCell
Images that are representative of nuclear and cytoplasmic sections were selected and Z projected to create nuclear and cytoplasmic single images. These were contrast adjusted and processed using DeepCell to generate a label matrix file of segmentation masks for each ROI (cropped version shown for illustrative purposes)

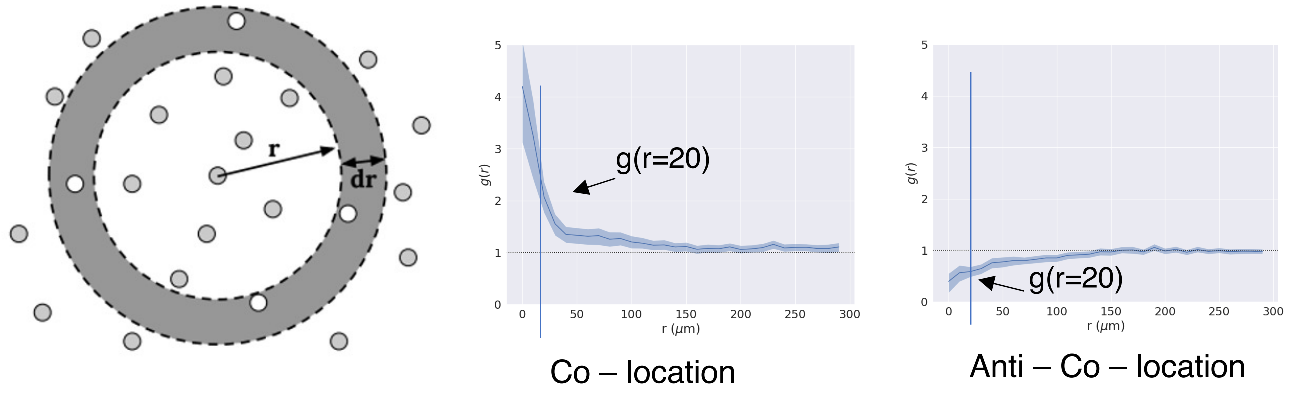

Supplementary Fig. 14. Pair correlation function

Derivation of the pair-correlation function showing demonstrative cross-PCF profiles for co – location and anti – co- location

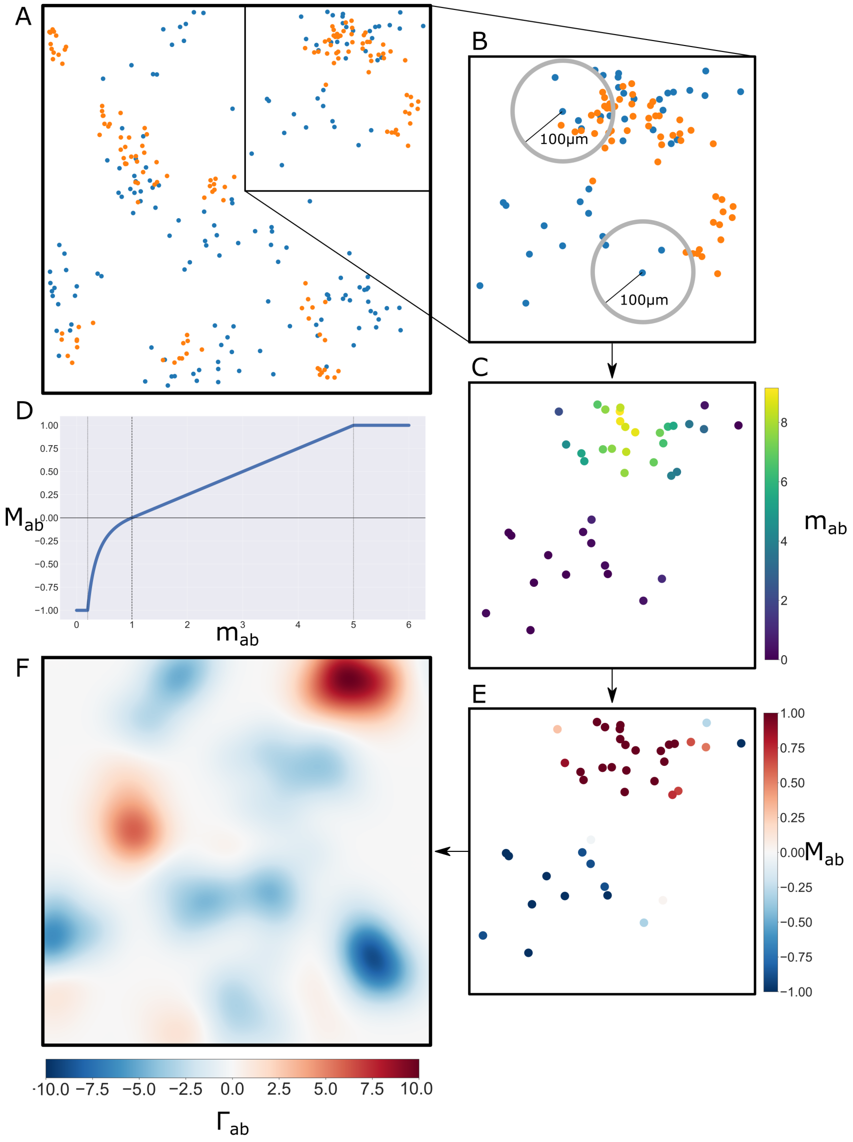

Supplementary Fig. 15. Process of creating a topographical correlation map (TCM).

A. Locations of points with labels A (blue) and B (orange) are identified within the region of interest.
B. The number of cells of type B within a 100µm radius of each cell of type A is calculated. This number is compared with the number of cells of type B that would be found within this radius under complete spatial randomness (CSR) to calculate the mark m_ab.
C. Cells of type A coloured according to the mark m_ab.
D. Sketch showing how the normalised mark M_ab depends on the mark m_ab. The magnitude of M_ab describes the strength of interaction while the sign indicates whether the interaction is correlation (M_ab> 0) or anti-correlation (M_ab<0).
E. Cells of type A coloured according to the normalised mark M_ab.
F. A kernel (Gaussian) of height M_ab is centred on each cell of type A. We sum the kernels to construct the topographical correlation map Γ_ab. High values of Γ_ab indicate close spatial proximity of cells of type B to cells of type A; large negative values indicate that there are few cells of type B in proximity to cells of type A.

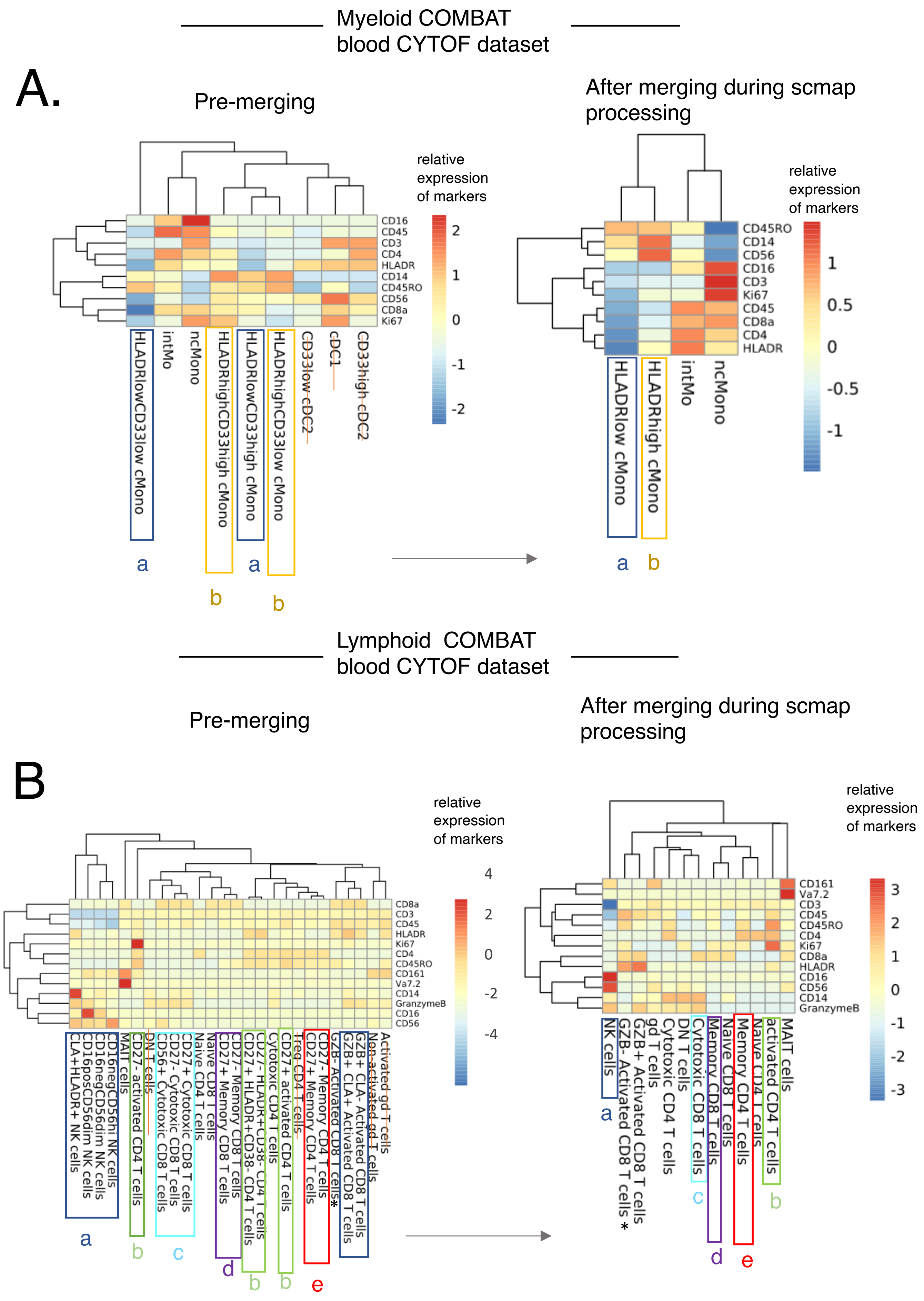
Myeloid COMBAT blood CYTOF dataset
A.
Pre-merging
After merging during scmap processing
relative expression of markers
CD16
CD45
CD3
CD4
HLADR
CD14
CD45RO
CD56
CD8a
Ki67
HLADRlowCD33low cMono
intMo
ncMono
HLADRhighCD33high cMono
HLADRlowCD33high cMono
HLADRhighCD33low cMono
CD33low cDC2
cDC1
CD33high cDC2
CD45RO
CD14
CD56
CD16
CD3
Ki67
CD45
CD8a
CD4
HLADR
HLADRlow cMono
HLADRhigh cMono
intMo
ncMono
a
b
Lymphoid COMBAT blood CYTOF dataset
Pre-merging
After merging during scmap processing
B
relative expression of markers
CD8a
CD3
CD45
HLADR
Ki67
CD4
CD45RO
CD161
Va7.2
CD14
GranzymeB
CD16
CD56
CLA+HLADR+ NK cells
CD16posCD56dim NK cells
CD16negCD56dim NK cells
CD16negCD56hi NK cells
MAIT cells
CD27- activated CD4 T cells
DN T cells
CD56+ Cytotoxic CD8 T cells
CD27- Cytotoxic CD8 T cells
CD27+ Cytotoxic CD8 T cells
Naive CD4 T cells
Naive CD8 T cells
CD27+ Memory CD8 T cells
CD27- Memory CD8 T cells
CD27+ HLADR+CD38- CD4 T cells
CD27- HLADR+CD38- CD4 T cells
Cytotoxic CD4 T cells
CD27+ activated CD4 T cells
Treg CD4 T cells
CD27+ Memory CD4 T cells
CD27- Memory CD4 T cells
GZB- Activated CD8 T cells*
GZB+ CLA+ Activated CD8 T cells
GZB+ CLA- Activated CD8 T cells
Non-activated gd T cells
Activated gd T cells
a
b
c
d
e
CD161
Va7.2
CD3
CD45
CD45RO
CD4
Ki67
CD8a
HLADR
CD16
CD56
CD14
GranzymeB
NK cells
GZB- Activated CD8 T cells *
GZB+ Activated CD8 T cells
gd T cells
Cytotoxic CD4 T cells
DN T cells
Cytotoxic CD8 T cells
Memory CD8 T cells
Naive CD8 T cells
Memory CD4 T cells
Naive CD4 T cells
activated CD4 T cells
MAIT cells

Supplementary Fig. 16. Composition of final merged clusters from CYTOF data in COMBAT for *scmap* matching to lung clusters of interest

A. COMBAT[6] cell clusters or 'centroid' (centroid is defined in scmap as a vector of the median values of expression for each gene) from CYTOF analysis of blood in all COVID-19 patients (n=64) showing myeloid clusters - before merging on left and after merging on right.
B. COMBAT cell clusters ('centroid') from CYTOF analysis of blood in all COVID-19 patients (n=64) showing lymphoid clusters - before merging on left and after merging on right.
In both A and B, coloured boxes on left correspond to the ones on right panel. Scored out clusters on left panel were removed as described in Methods. Those without boxes were not merged. Our eventual cell cluster of interest, GZB- activated CD8 T cells is marked with an asterisk, and it was not merged.
