# Supplementary material for: Single cell spatial analysis reveals inflammatory foci of immature neutrophil and CD8 T cells in COVID-19 lungs
Source: Nat Commun. 2023 Nov 8;14:7216. doi: 10.1038/s41467-023-42421-0 (PMC10632491; doi:10.1038/s41467-023-42421-0)
Supplement: Supplementary file 3 — Description of Additional Supplementary Files [file 41467_2023_42421_MOESM3_ESM.pdf]

Description of Additional Supplementary Files

Supplementary Data 1. Reagents, antibody panels and dilutions used in the paper
