# Supplementary material for: Single cell spatial analysis reveals inflammatory foci of immature neutrophil and CD8 T cells in COVID-19 lungs
Source: Nat Commun. 2023 Nov 8;14:7216. doi: 10.1038/s41467-023-42421-0 (PMC10632491; doi:10.1038/s41467-023-42421-0)
Supplement: Supplementary file 5 — Reporting Summary [file 41467_2023_42421_MOESM5_ESM.pdf]

nature portfolio

Corresponding author(s): Prof Ling Pei Ho

Last updated by author(s): Sep 8, 2023

Reporting Summary

Nature Portfolio wishes to improve the reproducibility of the work that we publish. This form provides structure for consistency and transparency in reporting. For further information on Nature Portfolio policies, see our Editorial Policies and the Editorial Policy Checklist.

Statistics

For all statistical analyses, confirm that the following items are present in the figure legend, table legend, main text, or Methods section.

n/a	Confirmed	
☐	☒	The exact sample size (*n*) for each experimental group/condition, given as a discrete number and unit of measurement
☐	☒	A statement on whether measurements were taken from distinct samples or whether the same sample was measured repeatedly
☐	☒	The statistical test(s) used AND whether they are one- or two-sided<br>*Only common tests should be described solely by name; describe more complex techniques in the Methods section.*
☐	☒	A description of all covariates tested
☐	☒	A description of any assumptions or corrections, such as tests of normality and adjustment for multiple comparisons
☐	☒	A full description of the statistical parameters including central tendency (e.g. means) or other basic estimates (e.g. regression coefficient) AND variation (e.g. standard deviation) or associated estimates of uncertainty (e.g. confidence intervals)
☐	☒	For null hypothesis testing, the test statistic (e.g. *F*, *t*, *r*) with confidence intervals, effect sizes, degrees of freedom and *P* value noted<br>*Give P values as exact values whenever suitable.*
☒	☐	For Bayesian analysis, information on the choice of priors and Markov chain Monte Carlo settings
☒	☐	For hierarchical and complex designs, identification of the appropriate level for tests and full reporting of outcomes
☒	☐	Estimates of effect sizes (e.g. Cohen's *d*, Pearson's *r*), indicating how they were calculated

Our web collection on statistics for biologists contains articles on many of the points above.

Software and code

Policy information about availability of computer code

Data collection	Imaging mass cytometry (IMC) data were acquired using the Hyperion imaging system (Standard BioTools) following manufacturer recommended procedure. The selected regions of interest were laser ablated at 200Hz, and data visualized using a commercial acquisition software (Hyperion imaging system v7.0.8493.0, Standard BioTools)
Data analysis	All IMC MCD files were inspected for the quality of staining (MCD viewer V. 1.0.560.2 Standard BioTools).<br>A customized pipeline SpOOx (Spatial Omics Oxford Pipeline) was used to process all data through MCD file conversion, segmentation, signal extraction, clustering and spatial analysis.<br>The complete code for the Spatial Omics Oxford pipeline is available as a GitHub repository under the GPL license: https://github.com/Taylor-CCB-Group/SpOOx. In addition, the SpOOx pipeline has been deposited at Zenodo (https://zenodo.org/record/8320986).<br><br>Visualization and final analysis of the data were achieved by a bespoke platform called Multi-Dimensional Viewer (MDV)<br>The Multi-Dimensional Viewer code is available under the GPL license: https://github.com/Taylor-CCB-Group/MDV. This package has also been deposited at Zenodo (https://zenodo.org/record/8324918). Tutorials and further documentation for the use of MDV are available on the web site: https://mdv.molbiol.ox.ac.uk/

For manuscripts utilizing custom algorithms or software that are central to the research but not yet described in published literature, software must be made available to editors and reviewers. We strongly encourage code deposition in a community repository (e.g. GitHub). See the Nature Portfolio guidelines for submitting code & software for further information.

Data

Policy information about availability of data

All manuscripts must include a data availability statement. This statement should provide the following information, where applicable:

- Accession codes, unique identifiers, or web links for publicly available datasets
- A description of any restrictions on data availability
- For clinical datasets or third party data, please ensure that the statement adheres to our policy

Data availability
The spatial mass cytometry dataset (MCD) files and results of analysis by the Spatial Omics Oxford pipeline are available at https://doi.org/10.5281/zenodo.6513508. The analysis results are also presented as a dynamic online resource in Multi-Dimensional Viewer (MDV) (https://mdv.molbiol.ox.ac.uk/projects/hyperion/6567). All source data are found in https://doi.org/10.5281/zenodo.6513508; and also within the hyperion 6567 project in the MDV link. Specific source data for graphs are also provided in Source Data File.

Human research participants

Policy information about studies involving human research participants and Sex and Gender in Research.

Reporting on sex and gender	Sex is reported in the patient information table (Supplementary Table 1)
Population characteristics	The characteristics of the included patients are presented in Supplementary Table 1
Recruitment	Patient samples were obtained from the University of Navarra, Spain and comprised those patients who died in hospital after admission with COVID-19. Healthy lung controls were obtained from the Oxford Centre for Histopathology Research and the Oxford Radcliffe Biobank based at the Oxford University NHS Hospitals Foundation Trust.
Ethics oversight	Ethics Committee of the University of Navarra, Spain (2020.192p) Oxford A South-Central NHS REC (19/SC/0173)

Note that full information on the approval of the study protocol must also be provided in the manuscript.

Field-specific reporting

Please select the one below that is the best fit for your research. If you are not sure, read the appropriate sections before making your selection.

☒ Life sciences ☐ Behavioural & social sciences ☐ Ecological, evolutionary & environmental sciences

For a reference copy of the document with all sections, see nature.com/documents/nr-reporting-summary-flat.pdf

Life sciences study design

All studies must disclose on these points even when the disclosure is negative.

Sample size	We used formalin-fixed paraffin-embedded (FFPE) lung sections from a cohort patients (n=12) who died from PCR-positive COVID-19 pneumonitis from one hospital (University of Navarra, Spain). Healthy lung sections were obtained from patients undergoing lobectomy for early, isolated lung cancer ('HC') lungs (n=2) were used as comparators; obtained from the Oxford Radcliffe Biobank (Oxford University Hospitals NHS Foundation Trust, UK). A statistical method was not used for sample size determination. This is the first time these spatial statistical methods have been applied to imaging mass cytometry (IMC) data sets hence the effect size and variation in the samples could not be determined prior to these experiments. Therefore, we based our sample size and sample collection area on lung imaging and omics technology experience as well as published imaging mass cytometry datasets. A total of 677,623 segmented cells were acquired from our samples for analysis which is comparable with other published imaging mass cytometry analyses. In addition another study with some similarities (DOI: 10.1038/s41586-021-03475-6) also used 12 individuals.
Data exclusions	Patients without PCR+ results for nucleocapsid (N) and/or envelope protein (E) in lung or liver tissue sample and those with evidence of bacterial culture from blood and lung within 3 days of death.
Replication	For all 12 patients, one lung sample was obtained per patient. Each lung sample generated one lung section. From each lung section, 2-3 replicate ROIs were obtained giving a total of 26 ROIs in COVID samples. All samples were stained once using the 37-plex IMC panel. There were 4 individual patient in each histopathology grouping (ALV, DAD and OP).THese provided a final total of n=10 ROIs altogether from ALV state, n=8 for DAD and n=9 for OP. Key findings were validated using immunoflorescence imaging. All immunofluorescence staining was performed once in lung samples from n=3 different patients. Due to the size, cost and analysis time, the imaging mass cytometry experiments were conducted only once.
Randomization	All 12 COVID lung samples and 2 HC samples were stained once with the 37 plex IMC panel over 3 different days. The groups for each staining day were organized so they contained comparable COVID and HC samples, and even distribution of ALV, DAD and OP COVID samples/ROIs. The order of data acquisition after staining on the Hyperion IMC system was randomized.

Blinding	All data acquisition was conducted blinded to associated clinical data. The researcher who stained the samples for IMC was blinded to the experimental group, as were the researchers who ran the samples for IMC. The researchers who stained the samples for IF and viral protein expression were blinded to the categorisation of samples (COVID vs HC; and ALV, DAD v OP). The bioinformaticians and data scientists who developed the pipeline and examined the data were blinded to the categorisation of samples. The data analysis pipeline was performed with no knowledge of sample categories.

Reporting for specific materials, systems and methods

We require information from authors about some types of materials, experimental systems and methods used in many studies. Here, indicate whether each material, system or method listed is relevant to your study. If you are not sure if a list item applies to your research, read the appropriate section before selecting a response.

Materials & experimental systems

n/a	Involved in the study
☐	☒ Antibodies
☒	☐ Eukaryotic cell lines
☒	☐ Palaeontology and archaeology
☒	☐ Animals and other organisms
☐	☒ Clinical data
☒	☐ Dual use research of concern

Methods

n/a	Involved in the study
☒	☐ ChIP-seq
☒	☐ Flow cytometry
☒	☐ MRI-based neuroimaging

Antibodies

Antibodies used	Details on antibody targets and clones are presented in Supplementary Data (Reagents) . All antibodies are commercially available and have been deposited in the Antibody Registry with deposition numbers included.<br>All the primary and secondary antibodies (with supplier name, catalog number, clone name and lot number, as applicable), are listed together with their final dilution. As there are >100 antibodies, we have not pasted it here.
Validation	Antibody clones were selected which had previously been published and validated in IMC studies as well as antibodies validated and frequently utilized for immunofluorescence or immunohistochemistry studies with human FFPE tissues. All Fluidigm antibodies used in this study are Maxpar® antibodies developed and optimized for use with the Hyperion™ Imaging System with formalin-fixed, paraffin-embedded (FFPE) human tissue sections. Further information on manufacture validation and use of clones in previous publications is available through the antibody registry https://www.antibodyregistry.org/ via the unique identifier for each clone listed in the antibody tables.<br><br>Staining validation for IMC markers was performed in healthy control lung as well as in some COVID-19 infected lung (Supplementary Fig. 5,6 and 12) as well as tonsil and bone marrow. Key markers were also validated using immunofluorescence.<br>During optimization, checks were performed to determine (i) mutually exclusive expression pattern were found in key immune and structural lineage markers (ii) appropriate sub-cellular location expression of the marker, iii) biologically coherent co - expression of markers. For further validation, we also examined if structural cell identities defined by IMC lineage marker expression were compatible with cell morphology and location on the corresponding H and E image.

Clinical data

Policy information about clinical studies

All manuscripts should comply with the ICMJEguidelines for publication of clinical research and a completedCONSORT checklist must be included with all submissions.

Clinical trial registration	This study was not part of a clinical trial
Study protocol	This study was not part of a clinical trial, hence not applicable
Data collection	Data was collected by perusal of the clinical and pathological records of the included patient samples
Outcomes	No primary or secondary outcome was defined
